# Politics embodied: How politics shapes and is shaped by the bodily experience of emotions

**DOI:** 10.1073/pnas.2534895123

**Published:** 2026-05-11

**Authors:** Andrea Vik, Alejandro Galvez-Pol, Sohee Park, Manos Tsakiris

**Affiliations:** ^a^Department of Psychology, Royal Holloway, University of London, Surrey TW20 0EX, United Kingdom; ^b^Centre for the Politics of Feelings. School of Advanced Study, University of London, London WC1 7HU, United Kingdom; ^c^Department of Psychology, University of the Balearic Islands, Mallorca 07122, Spain; ^d^Active Cognition, Embodiment, and Environment Lab, Cognition and Society Research Unit, University of the Balearic Islands, Mallorca 07122, Spain; ^e^Department of Psychology, Vanderbilt University, Nashville TN 37240

**Keywords:** political emotions, bodily sensations, political attitudes and behavior, emBODY tool

## Abstract

Political emotions are widely acknowledged as key drivers of political participation and polarization. Yet while it is well established that political emotions matter, far less is known about how they are felt and represented in the body. Across a preregistered, nationally representative study (*N* = 992), we introduce an embodied approach to political emotion using the validated emBODY-tool, which allows participants to map where in the body they experience sensations when feeling canonical emotions (e.g., anger) and their political counterparts (e.g., “political anger”). Specifically, we address three questions: 1) how political emotions are embodied and differ from their nonpolitical counterparts, 2) whether political dispositions influence how these political emotions are embodied, and 3) how their embodied experience interacts with political dispositions in explaining political attitudes and behavior. Pixelwise bodily sensation maps and aggregated “embodied impact” metrics show that political anger, anxiety, depression, disgust, and hope do not merely mirror their canonical forms, but take on distinct bodily patterns. Political ideology, but not political sophistication, modulates these bodily experiences, with Democrat-leaning participants reporting more intense sensations for negative political emotions, suggesting the presence of “ideological bodies.” Crucially, political participation is not explained by how intensely people report feeling emotions, but is instead closely linked to how strongly these emotions are embodied in the body. Together, our findings underscore the body’s central role in democratic engagement by showing how political contexts shape embodied emotional experience and how these embodied experiences shape politics and democracy.

## Significance

Emotions are central to politics, and the body is central to emotional experience, yet we know little about how political emotions are actually felt and represented in the body. This study shows that political emotions, such as political anger, anxiety, depression, disgust, and hope, take on distinct bodily patterns rather than simply mirroring how the same emotions are experienced in everyday life. These patterns shape how people engage with democracy. Using body-mapping methods with nearly 1,000 participants, we demonstrate that these political emotions are embodied differently according to people’s ideology and that they meaningfully predict political participation. Political emotions are therefore deeply embodied and distinctively motivate action, emphasizing the body’s importance for politics and democratic participation.

Politics is emotional. From politicians to voters, anger, anxiety, depression, disgust, and even hope permeate almost every aspect of the political landscape. Recently, political science, psychology, and neuroscience have undergone an “affective turn,” accepting that emotions are essential for political engagement, mobilization, and the formation of political identities (1–3). At the same time, these very emotions contribute to rising polarization, populism, and democratic disenchantment (4), and politics itself may be a substantial source of emotional dysregulation (5). As many as 40 % of Americans report that politics is a significant source of stress (5) and 77% cite the nation’s future as a major source of stress according to the American Psychological Association 2024 Stress in America®poll. For better or worse, how we feel about politics greatly influences our political reality.

While it is clearly acknowledged that political emotions matter, how these emotions are felt and experienced, not just cognitively, but physically, remains largely unexplored. In political science, emotions are often treated as disembodied mental states that can be accessed and measured simply by asking people to rate on a scale how intensely angry, anxious, or hopeful. Other times, such self-reports are complemented by psychophysiological measures (6). Together, these methods often yield inconsistent results as there is no one-to-one mapping between a given physiological variable and the conscious experience of a particular emotion (7–9). This reflects a broader limitation in the present understanding of political emotions: Embodiment lies at the core of emotional experience, as emotions are felt through the body as lived, interoceptive states (10, 11). As it is difficult to make a direct inference from psychophysiological reactivity to self-reported emotions, we need an expansion into how these bodily changes are experienced by people, not just in terms of their cognition or physiology but also in terms of their bodily experience (i.e., what does it feel like for my body to be in a state of political anger). By bodily experience, we refer to the self-aware, somatosensory feeling of emotion, how emotions are lived and sensed in the body, rather than disembodied mental states or purely physiological signals.

Here, we address this gap by focusing on the embodied experience of political emotions. Understanding whether political emotions are embodied differently from everyday emotions is important for several reasons. First, it allows us to move beyond largely untested assumptions in (political) psychology. Competing theories of emotion imply different expectations; classic basic emotion accounts would predict limited differentiation between political and nonpolitical emotions, whereas constructivist approaches emphasize context sensitivity and therefore allow for greater divergence (11). There are perhaps several nonmutually exclusive ways in which political emotions may differ from their nonpolitical counterparts: Their contextual and socially embedded construction, their ambiguity and complexity, and the individual variability as political meaning is filtered through ideology and identity (12). Political emotions are often discussed (and perhaps experienced) in abstract, collective, and morally charged contexts, which may alter how interoceptive signals -essential for experiencing emotions- are interpreted and organized. Whether these features translate into domain-specific embodied profiles, or whether political emotions resemble their everyday counterparts, remains an empirical question. Clarifying this distinction matters not only for theories of emotion but also normatively, because political emotions can both mobilize or demobilize democratic action. Differences in how anger, anxiety, or hope are felt—in intensity, scope, or bodily manifestation—may shape political behavior in ways that everyday emotions do not. Identifying unique embodied profiles could offer insights into how and why emotions are linked to political ideology and if and how political conflicts are perceived as visceral and polarizing. An embodied perspective is therefore crucial for both psychology and political science as it can clarify the body’s role in political engagement and democratic participation.

We adopt and extend the emBODY-tool that can be used to produce somatotopic maps, a topographical visualization of people’s emotional experience (13). The emBODY-tool asks participants to indicate regions of increased or decreased bodily sensations associated with distinct emotions by coloring in body silhouettes. Importantly, these bodily sensation maps (BSM) align with physiological, meta-analytic neuroimaging evidence, and cross-cultural findings (e.g., anxiety activates the chest and head, depression deactivates the limbs) (14, 15), and have been previously used successfully in relation to social issues (16), aesthetics (17, 18), social emotions (19), and moral violations (20). Importantly, bodily maps are not mere reflections of physiological monitoring, but expressive interfaces between brain, body, and conceptualization, relying on interoceptive sensations rather than just verbal articulation (21). By extending this method into the study of political emotions, we can bring to light their embodied topography (i.e. “where and what” of peoples emotional experience) and their embodied impact (i.e. the “breadth and depth” of people’s embodied experiences), derived from key metrics such as the diffusion, size, and intensity of bodily sensations (22, 23). In doing so, we can start to understand the political dimension of our embodied experience and its consequences for our politics.

Our first research question (RQ1) asks how political emotions—anger, anxiety, depression, disgust, and hope—are experienced in the body compared to their nonpolitical counterparts, in terms of 1) embodied topography (body–sensation maps) and 2) embodied impact (size, intensity, and spread). These five emotions span a theoretically rich spectrum central to political life. Each carries distinct motivational dynamics: Anger mobilizes and polarizes (1, 4); anxiety heightens vigilance (24); disgust moralizes and divides (25); depression demobilizes and can channel frustration into conspiratorial or violent tendencies (26, 27); and hope sustains engagement yet may legitimize aggression when linked to victory (28, 29). These emotions differ systematically in valence, arousal, and bodily profile (14), making them ideal for testing whether political contexts alter their embodied signatures. If political emotions feel distinct from canonical emotions, this would suggest that they are not merely general emotions applied to political targets but a qualitatively different class of affective experience—one with direct implications for democratic engagement and polarization.

Our second research question (RQ2) examines how individual political differences—specifically, political sophistication and ideological orientation—shape the embodied experience of political emotions, as reflected in their 1) embodied topography (body–sensation maps) and 2) embodied impact. These factors are central to understanding emotional responses to politics, yet evidence remains mixed. Politically sophisticated individuals often show stronger emotional engagement with political stimuli (1, 30, 31), though this pattern varies across emotions (32) and is not consistently mirrored in physiological data (33). Findings on ideology are likewise inconsistent: While conservatives sometimes exhibit heightened fear and disgust sensitivity (34), such effects may depend more on extremity or context than ideology itself. Physiological reactivity to threat and disgust shows limited partisan differentiation (35), whereas self-reported emotions do, influencing downstream political behavior (8, 25). These inconsistencies highlight the unresolved relationship between political dispositions and emotional processes. By adopting an embodied framework, we test whether political sophistication (a composite of political knowledge and interest) and ideology (proxied by partisan leanings in the United States), shape not only how people report feeling political emotions but how they experience them in their bodies.^*^

Last, our third research question (RQ3) examines how embodied emotional experiences interact with political dispositions to shape key political outcomes, including political participation and affective polarization. Previous research shows that emotions can drive both political participation (1) and affective polarization (37), yet emotions rarely operate in isolation when explaining political attitudes and behavior. Some studies suggest that certain emotions increase participation primarily among politically sophisticated individuals (1, 31), whereas others find that anger, in particular, mobilizes those with low prior political involvement (32). Stronger partisan identities also heighten emotional reactivity and exacerbate affective polarization (38, 39), especially for emotions such as anger, anxiety, and disgust (37). Together, these findings highlight that emotional influences on political behavior are complex and potentially nonlinear—a dynamic we seek to clarify through an embodied framework.

To address these questions, we conducted a preregistered within-subjects experiment on Prolific with a representative sample (N=992).^†^ The design and preprocessing are illustrated in Fig. 2 (see also *Materials and Methods* and *SI Appendix*, section A). Participants completed a nonpolitical emBODY task, indicating where they felt bodily activations and deactivations for five everyday emotions by coloring two body silhouettes, a method shown to produce reliable bodily sensation maps (13, 14). They also completed a political emBODY task in which, for each emotion, they selected a relevant political issue, rated the emotion’s intensity, and mapped the associated bodily sensations. The emBODY-tool protocols are further explained and illustrated in *SI Appendix*, section A3. These maps were processed into topographical body–sensation maps and a corresponding aggregate measure of embodied impact, which serve as the basis for our analyses and allow us to answer our research questions.

## Results

### RQ1: Are Political Emotions Experienced Differently Compared to Canonical Emotions?

**Fig. 1.**
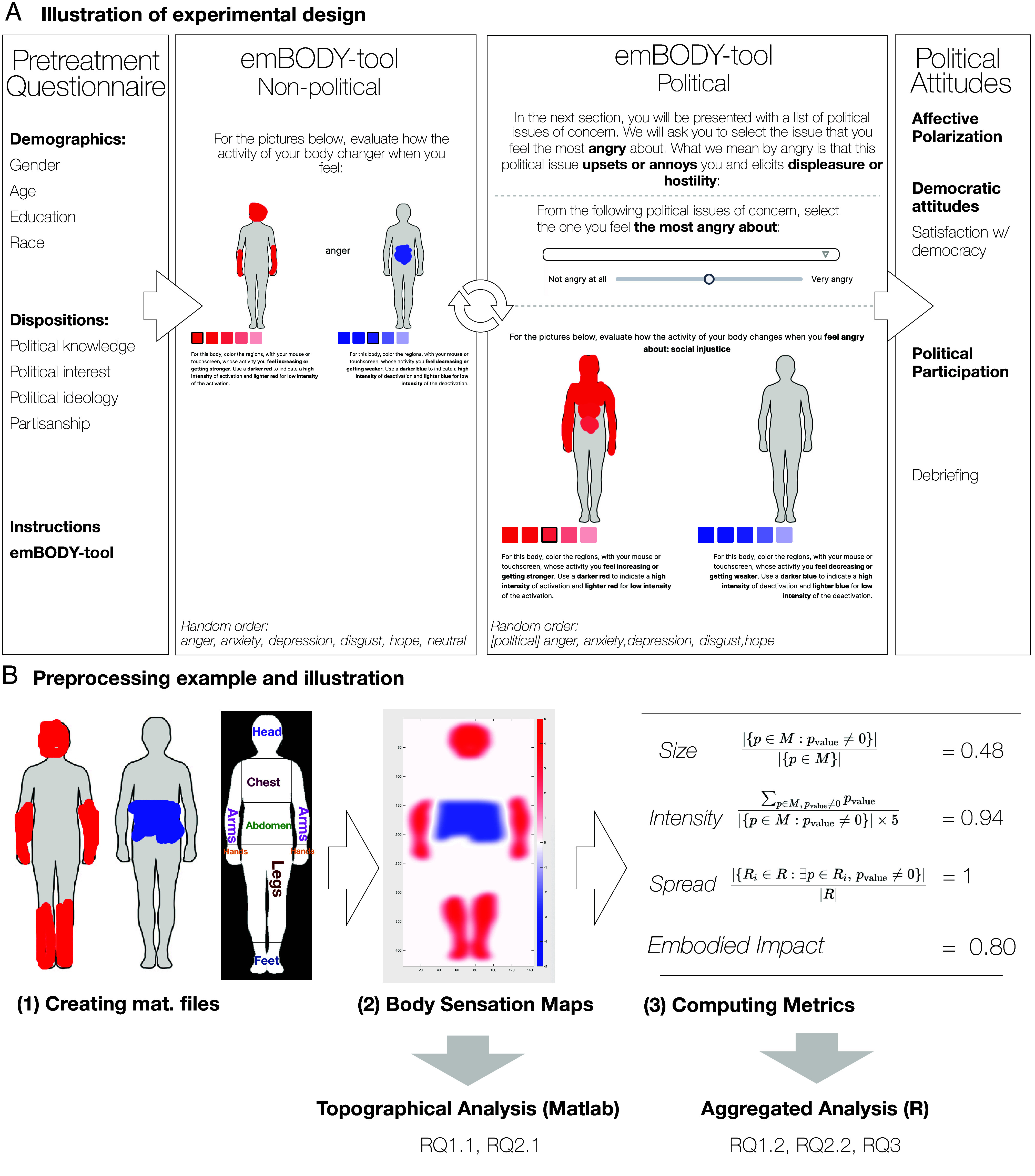
Bodily representations of political and nonpolitical emotions (*A*) The results of the one-sample *t* tests (top two rows) and pixelwise paired *t* tests (third row) for the subjectwise BSMs. In the top two rows, the body maps from the mass univariate pixelwise *t* tests show regions whose activation increased (warm colors) or decreased (cool colors) when feeling each emotion(BH–FDR corrected P<0.05). The third row shows pixelwise paired *t*-tests comparing political vs. nonpolitical conditions in which red colors indicate where political emotions had significantly higher values, and blue shows where political emotions had significantly lower values (BH–FDR corrected P<0.05). (*B*) Violin plots illustrating repeated-measures ANOVA results. Each pair of violins (e.g., Anger vs. Polanger) includes mean values and 95% CIs; lines above indicate BH-corrected *P*-values for pairwise comparisons.

Pixelwise analyses of the bodily–sensation maps (BSMs) revealed clear, emotion-specific differences between all political and nonpolitical conditions (Fig. 1*A*). Political anger differed from nonpolitical anger, with 28.2% of body-map pixels showing significant differences following false discovery rate (FDR) correction (|*t*| ≥2.46; 8,948 pixels), primarily in the arms. Anxiety showed only small but reliable differences (3.4%; |*t*| ≥3.15; 1,069 pixels), indicating that political and nonpolitical anxiety were largely similar, with slightly greater abdominal activation for the nonpolitical condition. The largest differences were observed for depression, where all pixels differed significantly between conditions (100%; |*t*| ≥2.74; 31,769 pixels); political depression was characterized by less deactivation and more widespread activation than nonpolitical depression. Disgust also showed extensive differences (83.1%; |*t*| ≥2.04; 26,384 pixels): The political disgust map was less sharply localized to the abdomen–esophagus region and more similar in overall shape to anger (cosine similarity = 0.99; see *SI Appendix*, Fig. S7 and section B1). Hope also differed reliably (68.7%; |*t*| ≥2.12; 21,816 pixels). Together, these results indicate that politicizing emotions produces significant and systematic differences in their embodied patterns of activations and deactivations rather than a uniform amplification or suppression.

**Fig. 2.**
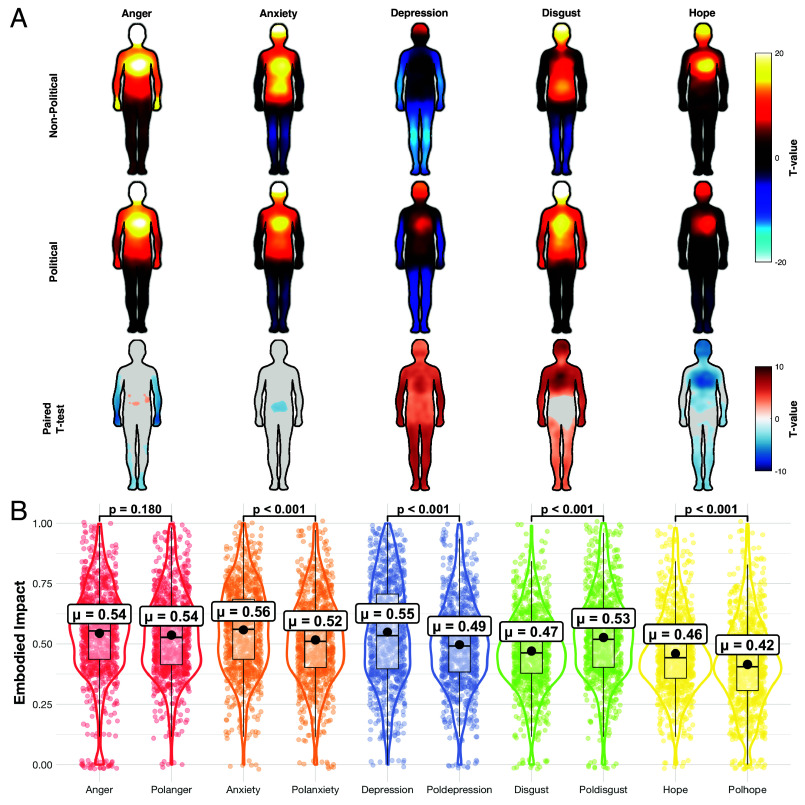
Experimental design and preprocessing. (*A*) Design. The preregistered within-subjects design included four parts: a pretask questionnaire, a nonpolitical emBODY protocol, a political emBODY protocol, and a posttask questionnaire. Participants mapped bodily activations (red) and deactivations (blue) for six nonpolitical emotions and, in a second task, selected political issues one for each political emotion, and they were asked to rate the intensity of this emotion, and last, mapped their bodily sensations for these political emotions. Task order was counterbalanced, and emotion order was randomized. (*B*) Preprocessing. For each bodily sensation map (BSM), an Otsu-thresholded mask isolated valid body pixels, and activation and deactivation images were converted from RGB values to numeric intensities (−5 to +5) and smoothed with a 0.5 Gaussian kernel. These were combined into subject-wise maps, from which we derived four metrics: size, intensity, ROI coverage, and a composite embodied impact score. All metrics were saved for subsequent analysis in R.

Aggregated analyses of the embodied impact metric closely paralleled the pixelwise results (Fig. 1*B*). Political and nonpolitical anger did not differ (mean difference = 0.008, 95% CI [−0.008,0.023], P=0.180), and equivalence testing supported a practically negligible effect (TOST, P<0.001). By contrast, nonpolitical anxiety, depression, and hope showed higher embodied impact than their political counterparts (all t(991)≥7.64, all P<0.001), and these differences were not equivalent under the preregistered bounds. Political disgust showed the opposite pattern, eliciting greater embodied impact than nonpolitical disgust (t(991)=−10.03, P<0.001, Δ=−0.056). Preregistered robustness checks (activation- vs. deactivation-only maps, removal of controls, cosine-similarity evaluation) reproduced the core finding: Politicization reshapes embodied experience in an emotion-specific way, with the largest shifts for depression, disgust, and hope, and comparatively small or negligible shifts for anxiety and anger (*SI Appendix*, section B1).

### RQ2: Does Political Sophistication or Political Partisanship Shape the Bodily Experience of Political Emotions?

**Fig. 3.**
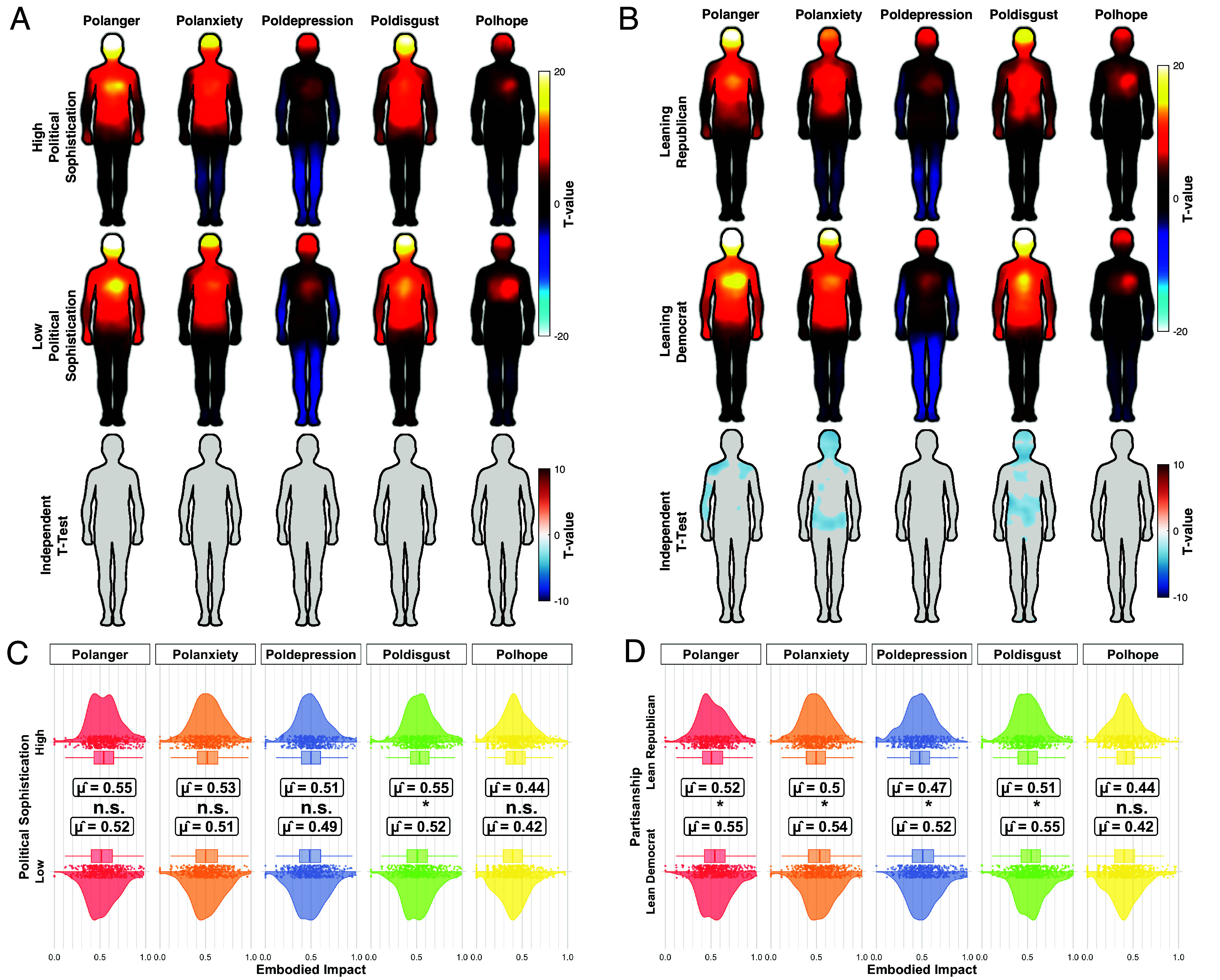
Influence of political dispositions on embodied political emotions. (*A* and *B*) The top two rows display the within-group pixel-wise *t* tests, and the third row shows between-group differences: Red indicates significantly higher values in one group compared to the reference group (high political sophistication and leaning republican). (*C* and *D*) Raincloud plots from a multilevel ANOVA examine individual differences across five political emotions (Polanger, Polanxiety, Poldepression, Poldisgust, Polhope), controlling for age, gender, ethnicity, and education. Half-violin densities with jittered participant points show the distribution of embodied impact for each political emotion (facets). Central error bars depict 95% CIs around estimated marginal means ($\hat{\mu }$).

We next asked whether these political bodily maps are sensitive to individual political differences (Fig. 3). Comparisons between participants with high vs. low political sophistication yielded very limited pixelwise differences: Across all five political emotions, maps were visually and statistically similar after FDR correction, indicating that the basic somatic organization of political emotions is largely shared across political sophistication levels (Fig. 3*A*). A mixed-model analysis of embodied impact confirmed this pattern. Political sophistication showed only a small main effect, F(1,990)=5.38, P=0.021, $\eta_{G}^{2}=0.003$, alongside a robust emotion effect, but the preregistered sophistication × emotion interaction was not significant. Pairwise tests were nonsignificant for the political emotions anger, anxiety, depression, and hope, whereas disgust showed a small, significant difference (*SI Appendix*, section B2). As a robustness check, we examined political sophistication by disaggregating it into political interest and political knowledge, substantially confirming our results (*SI Appendix*, Fig. S14 and section B3).

In contrast, political partisanship was associated with reliable differences in somatic experience (Fig. 3 *B* and *D*). Democrat-leaning participants (i.e., participants identifying with or leaning toward the Democratic Party on the partisan leaning scale) reported stronger bodily activation than Republican-leaning participants for the negative political emotions—political anger, anxiety, depression, and disgust—primarily in upper-torso and head regions. Aggregated embodied impact scores mirrored these maps. Democrats showed higher impact for political anger (M=0.547 vs. 0.514), political anxiety (0.531 vs. 0.488), political depression (0.518 vs. 0.461), and political disgust (0.541 vs. 0.498), all P<0.01 in the multilevel ANOVA, whereas political hope did not differ by partisanship. Notably, these partisanship differences were specific to embodied impact: Self-reported emotion intensity did not differ between Democrat- and Republican-leaning respondents (*SI Appendix*, section B2). We also find the same pattern when looking at nonpolitical emotions (*SI Appendix*, Fig. S10 and section B2), and also when we use ideological left–right placement as the predictor instead of partisanship (*SI Appendix*, Fig. S15 and section B3).

### RQ3: How Does the Embodied Impact of Political Emotions Shape Political Participation and Affective Polarization?

**Fig. 4.**
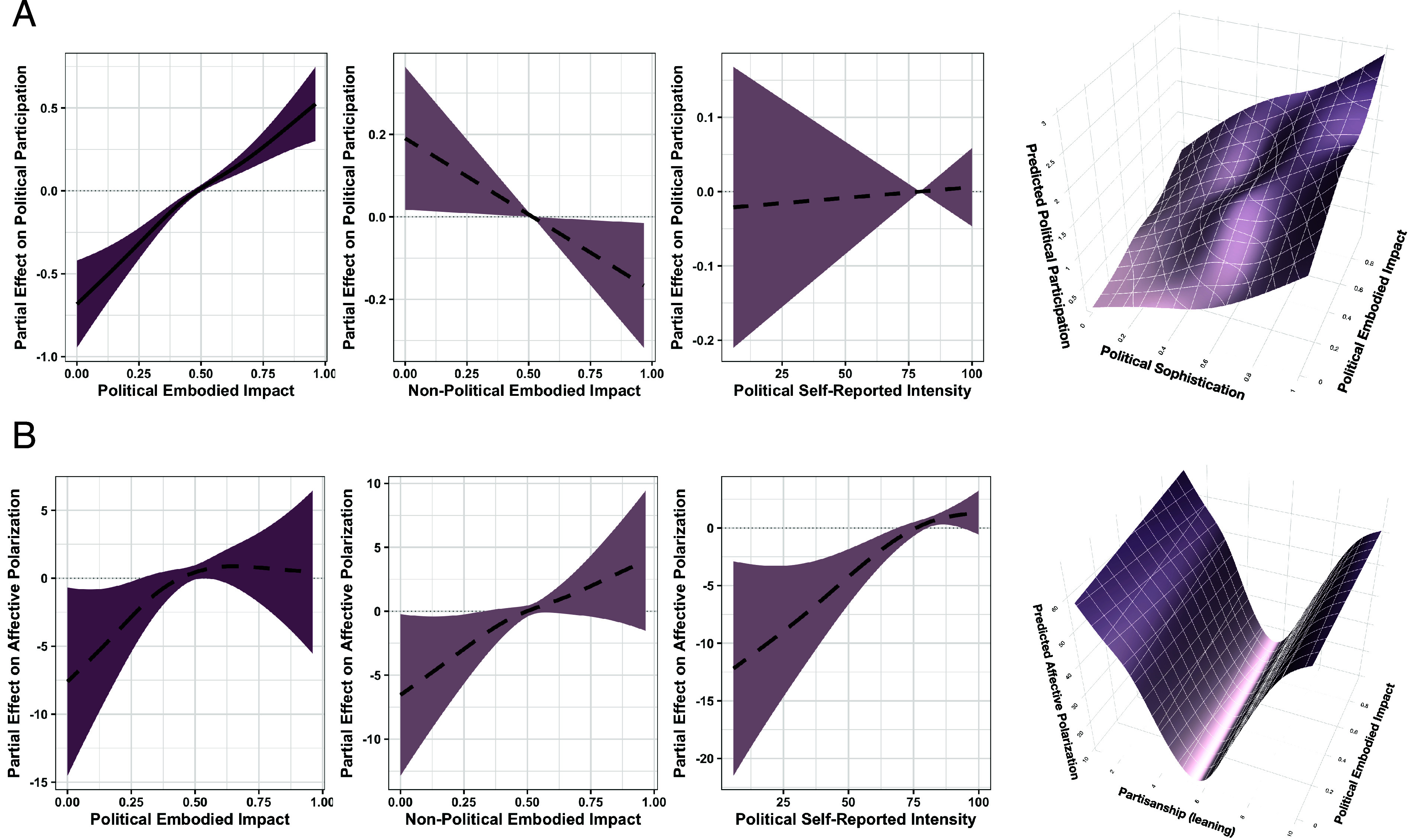
Partial and interactive effects from GAMs (REML) predicting (*A*) political participation and (*B*) affective polarization (full model output available in Appendix B3). Rows show smooth main effects of Political, Non-Political, and Self-Reported Emotional Impact; Political embodied impact is shown in darker color for emphasis. Lines represent smooth effects (no linearity assumed); shading denotes 95% CIs (local uncertainty); dashed lines indicate *P* 0.05, reflecting whether the smooth differs from zero overall. *Right* panels show preregistered interaction surfaces: Political Embodied Impact × Political Sophistication (*A*) and × Partisanship (*B*). All models control for age, gender, education, and ethnicity.

To address RQ3, we first examined main effects of embodied political impact, then applied the preregistered generalized additive models to test for nonlinear variation by individual differences. Higher embodied *political* impact was reliably associated with greater political participation (edf =1.86, F=5.31, P=0.0039). Importantly, neither embodied *nonpolitical* impact nor self-reported emotion intensity was a significant predictor (Fig. 4), suggesting an embodied and political specificity of the emotional impact that drives political participation. In the preregistered interaction, the embodied-impact × sophistication surface was not significant (edf =3.97, F=0.81, P=0.562), indicating that the participation effect of embodied political emotions was broadly similar across all sophistication levels. For affective polarization, neither the main-effect model (edf = 1.65, F=0.74, P=0.502) nor the preregistered interaction (edf =1.01, F=0.18, P=0.678) yielded significant effects of embodied impact; polarization was instead driven by partisan strength (full model specifications and preregistered robustness checks are reported in *SI Appendix*, section B3).

## Discussion

We aimed to address a fundamental scientific question about the nature of political emotions, but also a pressing societal question on the role that emotions and their embodiment play in politics. In short, we find that politics changes how we feel certain emotions in our bodies, and that the embodied experience of these political emotions matters for politics.

### Reframing Political Emotions.

We first asked whether political emotions are embodied differently from canonical emotions experienced in everyday life. Our findings suggest that once some emotions are given a political context, their bodily experience changes. Anxiety shows a distinct embodied profile, suggesting that politics evokes a qualitatively different kind of worry, one that perhaps is centered more on collective uncertainty and vigilance rather than personal threat (40). Depression appears less like a state of shutdown and more activated when it is politically directed, suggesting that depression toward political targets may mobilize rather than demobilize, in line with emerging evidence that political forms of despair can connect to engagement (27). Hope shows weaker bodily activation in political contexts, perhaps reflecting how its motivational force becomes split between aspirational and adversarial orientations (29), muting the energizing quality typically associated with hope. For anger, we find only modest differences between contexts, and the similarities in their bodily experience can help explain why anger retains a common mobilizing core that can easily be recruited into politics, as both everyday and political anger can drive political action and judgment (41, 42). Last, for disgust, the shift toward an anger-like pattern (cossim = 0.99, see *SI Appendix*, section B1) fits the view that political contexts moralize disgust, transforming it from pathogen avoidance into partisan defense (25). These embodied patterns of anger and disgust also highlight the limited emotional granularity typical of affective experience, where multiple emotions can co-occur (10).

Our approach reveals how political contexts reshape the bodily experience of emotion. This aligns with emerging evidence that emotions in politics may follow different appraisal patterns and psychological dynamics than emotions in personal contexts (43). Almost every contemporary theory of emotion acknowledges that bodily sensations—such as a racing heart or a knot in the stomach, are central to the subjective experience of “feeling” emotion (11). Basic Emotion Theory assumes that canonical emotions such as anxiety have biologically fixed bodily “fingerprints,” whereas constructivist models view emotions as contextually constructed from interoceptive and conceptual processes (10). Constructivist theories thus contest that categories of emotions (like “anxiety”) are statistical groupings of variable instances, not fixed essences. “Anxiety” is a conceptual label humans use to interpret diverse brain–body events that share functional similarities, such as preparing for uncertainty or threat. When context shifts, for example, from interpersonal to political, so too do the bodily states and felt experiences.

### Individual Differences in Embodied Emotion: A Case of Ideological Bodies?

We next asked whether individual differences in political sophistication and partisanship shape the embodied experience of political emotions. While political sophistication did not influence the embodied experience of emotions, partisanship did.

While we find no evidence that political sophistication shapes the embodied experience of political emotions, this null effect should be interpreted cautiously. Prior work suggests that politically sophisticated individuals often report stronger emotional engagement with politics, although this varies across emotions and is not consistently mirrored in physiological evidence (30, 33). Our null findings may partly reflect restricted variance in this highly sophisticated sample, but they also lend support to recent arguments that measures of political sophistication require reconceptualization and may conflate distinct mechanisms. In particular, emotional engagement appears to be driven more by political interest and confidence than by factual knowledge, whereas sophistication may operate primarily through biased information processing and resistance to counterattitudinal messages (44, 45).

Partisanship appeared to influence how emotions, both political and nonpolitical, are embodied. Democrats or Democrat-leaning participants reported feeling negative emotions more somatically, though this association is correlational and may reflect the historical context of data collection (July 2025), when Democrats were “electoral losers” in the 2024 US Presidential Election and worried about the politics of their country (46). Yet similar differences in nonpolitical emotions (*SI Appendix*, section B2) suggest that ideological orientation may correspond to deeper, possibly dispositional, affective tendencies that may also resonate with partisan differences in mental health (47). These findings suggest that we may have ideological bodies, akin to the concept of ideological brains that reflect cognitive predispositions (48). Just like basic cognitive and perceptual processes relate to our ideological dispositions, so can basic interoceptive, affective, and emotional processes structure the bodily felt patterns through which political worldviews are lived and reproduced (49, 50).

### Political Embodiment and Political Behavior.

Last, we tested the explanatory value of the embodied experience of emotions, over and above that obtained by classic measures of political emotion. Our findings suggest that political participation is driven by how strongly these political emotions are embodied, rather than by how intensely people say they feel emotions, or by the embodied impact of the same emotions in a nonpolitical context. When political emotions like anger, anxiety, and disgust are physically experienced, they appear to mobilize individuals toward action, highlighting the embodied specificity of political engagement. Embodied in the sense that what matters is where and how emotions are experienced in the body, rather than an approximation of a self-reported overall intensity score that is an abstraction from the actual embodied feelings. And specificity insofar as it is the embodied experience of political emotions, rather than the embodied experience of emotions in general, that explains the variance in political participation measures. However, we did not find the same effect for affective polarization. Affective polarization may be driven by emotions directed specifically at groups rather than policy issues or by more stable identity-based dynamics rather than momentary embodied affect. These hypotheses highlight the need for more research into the affective and embodied roots of social identity and intergroup relations that could then explain how affective polarization emerges from the body and how it can affect our bodies and health (38, 50, 51).

In line with the theory of constructed emotion (52), bodily regulation and emotional meaning cannot be meaningfully separated; political action arises when bioregulatory states are experienced as politically relevant. Normatively, our study raises important questions about the role of the body in democratic life: If participation depends on how politics is felt, inequalities in affective or embodied responsiveness could shape who acts and whose voices are heard. Understanding the bodily dimensions of political emotions is therefore not only empirically important but also crucial for debates about affective justice and the conditions of democratic participation.

### Limitations and Directions for Future Research.

Several limitations should be acknowledged, though we view many as productive directions for future research.

Bodily maps capture graded topographies of felt activation and deactivation across the body; overlap along this embodied dimension does not rule out differentiation along other emotion-relevant dimensions not directly measured here (e.g., appraisal structure, temporal orientation, or social meaning). Relatedly, our analyses relied on aggregated indices rather than region-specific effects, which were beyond the scope of the present paper. Although emerging work (21) suggests that bodily localization reflects underlying mechanisms that differentiate emotional experience, there is currently limited theoretical development linking specific body regions (e.g., arms/hands vs. head or chest) to action tendencies vs. cognitive processing. Advancing this line of inquiry will require clearer theorization, novel hypotheses, and designs that incorporate relevant moderators (e.g., need for cognition). It may also be most tractable within specific political domains—for example, examining whether the localization of feelings about climate change predicts distinct forms of climate action. While we sought to compare political and nonpolitical embodied responses, achieving true equivalence in emotional intensity across domains is inherently difficult. Future studies could incorporate a third, affectively matched but nonpolitical condition (e.g., disgust from a physical contaminant) to more precisely isolate the role of political framing, though we note that such equivalence is conceptually challenging. Politicization itself may transform the meaning of emotions: Political disgust may acquire moral content, and political depression may differ qualitatively from its clinical counterpart. We view this as an opportunity to empirically test whether constructivist or essentialist models better account for such context-dependent transformations. Finally, our primary objective was empirical, but the findings underscore the need for a stronger theorization of emotion in political science and beyond.

The U.S. context may represent a strong test of our claims, given the salience of partisan identities and conflict, whereas political emotions may be weaker or more issue-specific in less polarized or multiparty systems. However, as polarization and media-driven political conflict intensify in many democracies, similar affective dynamics may increasingly generalize beyond the U.S. context. More comparative work is needed to test how political context and levels of polarization moderate the embodied experience of political emotions. Furthermore, because partisanship closely tracks ideology in the United States—but not necessarily elsewhere- comparative work should more carefully disentangle partisan and ideological bodies. Beyond context and political differences, unobserved individual differences, such as personality traits or chronic affective styles may contribute to bodily sensation patterns not fully captured in our models. Future research would benefit from including such psychological predispositions in its designs. Although our within-subject design identifies systematic embodied differences for political emotions, a stronger test of the causal mechanisms, particularly whether embodiment drives political engagement or reflects it, will require longitudinal experimental designs, given the bidirectional links between emotion and behavior.

### Conclusion.

That emotions matter for politics is well established (12), but we here show how politics both shapes and is shaped by the bodily experience of emotion and why this matters. Political emotions are, to a large extent, embodied in distinct ways from the embodiment of the same emotions in everyday life. Importantly, it is the embodied specificity of the political emotions that motivates political participation, emphasizing the body’s central role for politics and democratic engagement.

## Materials and Methods

### Ethics.

All procedures were approved by the Research Ethics Committee of Royal Holloway, University of London (Approval No. 312) and conducted in accordance with the Declaration of Helsinki. Participants provided informed consent prior to participation and were remunerated at £9 per hour (pro rata for the 15 to 20 min online session). The study involved no foreseeable risks or vulnerable populations.

### Participants and Sampling Plan.

Sample size was determined through a preregistered power analysis based on a pilot study (N=121), which estimated the within-subjects effect size for the primary 10-level repeated-measures ANOVA (f=0.41, $\overset{¯}{r}=0.50$). Simulations using generalized additive models (GAMs) indicated ≥99% power for small effects (d=0.20) at N=800; thus, we recruited N=1,021 U.S.-based participants via Prolific in July 2025 using the platform’s U.S. representative sampling framework to approximate population distributions for age, gender, ethnicity, and party identification.

Data-quality exclusions were preregistered. Participants were removed if they failed at least two of three attention checks, missed more than one comprehension question about the emBODY tool, or submitted unusable body maps (e.g., random or blank responses). Because all survey items were forced-response, there was no item-level attrition; the reduction from 1,021 to 992 participants reflects preregistered data-quality exclusions rather than participant dropout or nonresponse. The final analytic sample comprised 992 participants (median age = 46 y; 50% women; 63% White; 29% Republican, 32% Democrat, 39% Independent). Full sample characteristics appear in *SI Appendix*, section A2. All analyses were conducted on unweighted data.

### Design and Procedure.

The preregistered design used a within-subjects, counterbalanced structure with four phases: a) pretask questionnaire, b) nonpolitical emBODY protocol, c) political emBODY protocol, and d) posttask questionnaire (Fig. 2). Data collection was implemented on the Gorilla platform. The full preregistration is available at this OSF link.

#### Pretask questionnaire.

Participants reported demographic information and completed validated measures of political dispositions: ideology (10-point left–right scale, European Values Survey), partisanship (Pew Research), political interest (European Values Survey), and political knowledge (four factual items adapted from ANES). They then watched an instructional video explaining the emBODY tool and completed comprehension checks (*SI Appendix*, section A1).

#### Nonpolitical protocol.

Participants completed the emBODY task (13) for six canonical emotions—anger, anxiety, depression, disgust, hope, and neutral—presented in random order (see *SI Appendix*, section A3 for more details). For each emotion, participants indicated perceived activation (red) and deactivation (blue) across two body silhouettes (144 × 427 px) using a fixed brush size (18 px) and intensity scale (−5 to +5).

#### Political protocol.

Participants repeated the emBODY procedure for five political emotions—political anger, anxiety, depression, disgust, and hope (see *SI Appendix*, section A3 for the task). For each emotion, participants were first prompted to consider that specific emotion and then selected a political issue from a standardized list of contemporary topics (53) that they personally associated with feeling that emotion. They then rated the intensity of that emotion in response to the selected issue (0 to 100 scale) and painted the corresponding bodily activations and deactivations. This procedure allowed participants to anchor each political emotion to a personally salient political context while holding the emotional category constant across participants. Order of political and nonpolitical tasks was counterbalanced across participants.

#### Posttask questionnaire.

Participants reported affective polarization (feeling thermometers toward Democrats and Republicans), satisfaction with democracy, and recent political participation (protests, petitions, voting, and online advocacy; ANES items). Open-ended feedback was collected prior to debrief.

### Preprocessing and Derived Measures.

BSMs were processed in MATLAB 2023b (MathWorks). Binary masks (Otsu thresholding) defined valid body pixels; RGB intensity values were mapped onto a −5 to +5 numeric scale and smoothed with a 0.5 Gaussian kernel, following previous research (13). Activation and deactivation maps were combined into composite subject-wise BSMs. Four metrics were extracted per emotion: 1) **size**, proportion of body pixels painted (22, 23, 54); 2) **intensity**, mean normalized pixel value; 3) **spread**, proportion of seven anatomical regions containing ≥1 painted pixel; and 4) **embodied impact**, the standardized composite of these three dimensions. A detailed account of these metrics is available in *SI Appendix*, section A4. All indices were saved to a CSV for subsequent aggregated analyses in R 4.3.2 (R Core Team).

### Analytic Pipeline.

Analyses followed the preregistered two-tier analytical pipeline as illustrated in Fig. 2*B*.

1. *Topographical analyses* (MATLAB 2023b) comprised mass-univariate pixelwise *t* tests (one-sample against zero) for each emotion map and paired *t* tests comparing political and nonpolitical emotions. All maps were corrected for multiple comparisons using the Benjamini–Hochberg false-discovery rate (FDR).
2. *Aggregated analyses* (R 4.3.2) used derived embodied-impact scores to test preregistered hypotheses regarding mean differences and nonlinear associations. - Repeated-measures ANOVAs evaluated embodied-impact differences with *Emotion* as a within-subjects factor and, where relevant, political sophistication or partisanship as between-subjects factors. Pairwise contrasts were corrected using the Benjamini–Hochberg procedure, and equivalence was assessed via two one-sided tests (TOST; equivalence margin ±0.25). - Generalized additive models (GAMs; mgcv::gam, REML) predicted political participation and affective polarization from embodied impact. Tensor-product smooths (ti()) captured the preregistered nonlinear interactions with political sophistication or partisanship, while separate smooth terms modeled main effects.

As a minor deviation from the preregistration, all aggregated models (ANOVAs and GAMs) reported in the main text include sociodemographic control variables (age, gender, education, and ethnicity) to improve precision and comparability across analyses. The preregistered models without controls are presented in *SI Appendix*, section B, and additional clarifications of preregistered deviations appear in *SI Appendix*, section C. Robustness and alternative specifications are also detailed in *SI Appendix*, section B.

## Supplementary Material

Appendix 01 (PDF)

## Data Availability

All data and materials are available at OSF: https://osf.io/49byc/overview (55). The experiment, including all emBODY-tool protocols, was implemented using the Gorilla online research platform. The nonpolitical emBODY protocol is available at https://app.gorilla.sc/openmaterials/1189311 (56), and the political emBODY protocol is available at https://app.gorilla.sc/openmaterials/1189260 (57). Both protocols may be viewed, tested, and duplicated for use in future research.

## Footnotes

^*^In contemporary U.S. politics, partisanship and ideological self-placement are highly correlated and difficult to disentangle; we therefore use partisan leanings as our primary indicator of ideological orientation (36).

^†^All analyses were conducted on unweighted data.
