# Supplementary material for: Politics embodied: How politics shapes and is shaped by the bodily experience of emotions
Source: Proc Natl Acad Sci U S A. 2026 May 11;123(20):e2534895123. doi: 10.1073/pnas.2534895123 (PMC13187802; doi:10.1073/pnas.2534895123)
Supplement: Supplementary file 1 — Appendix 01 (PDF) [file pnas.2534895123.sapp.pdf]

# Supporting Information for

## Politics Embodied: How Politics Shapes and Is Shaped by the Bodily Experience of Emotions

**Andrea Vik, Alejandro Galvez-Pol, Sohee Park, and Manos Tsakiris**

**Andrea Vik.**
**E-mail: andrea.vik@rhul.ac.uk**

### This PDF file includes:

Figs. S1 to S16
Tables S1 to S8
SI References

## Appendix A: Supplementary Methods

**Appendix A1: Variables and Question Wordings.** The pre-treatment questionnaire included measures of demographic characteristics (age, gender, education, and ethnicity) and political dispositions (ideology, partisanship, political interest, and political knowledge). Demographic items were administered first in a fixed order, followed by political disposition items presented in randomized order. Exact item wording is reported below.

1. **Age:** Please enter your age. *(numeric response)*
2. **Gender:** What is your gender? *(choose one, random order)* Male / Female / Other–Nonbinary
3. **Education:** What is the highest level of education you have completed? *(choose one)*
    - No schooling completed
    - Some high school, no diploma
    - High school graduate (or GED)
    - Some college credit, no degree
    - Trade/technical/vocational training
    - Associate degree
    - Bachelor's degree
    - Master's degree
    - Professional degree
    - Doctorate degree
4. **Ethnicity:** Which of the following describes you? Select all that apply. *(random order)*
    - White
    - Hispanic or Latino
    - Black or African American
    - Native American or Alaskan Native
    - Hawaiian Native or Pacific Islander
    - Asian or Asian American
    - Middle Eastern or North African
    - Other
    - None of these
    - Prefer not to say
5. **Political Ideology:** In politics, people often speak of 'left' and 'right'. Where would you place yourself on a scale from 1 to 10, if 1 means 'left' and 10 means 'right'? *(slider:* $1 = left$, $10 = right$*) Source: European Values Survey*
6. **Political Partisanship:** Which of these best describes you today: Republican, Democrat, Independent, or something else? *(choose one, random order)* Democrat; Republican; Independent; Other.
7. **Partisan Leaning:** Do you lean more toward the Republican Party or the Democratic Party? *(slider:* $1 = DemocraticParty$, $10 = RepublicanParty$*) Source:* *Pew Research*
8. **Political Interest:** How interested are you in politics? *(slider:* $1 = notatallinterested$, $10 = veryinterested$*) Source: European Values Survey*
9. **Political Knowledge:**
    (a) How many years are there in one full term of office for a U.S. senator?
    (b) Which party held the most seats in the U.S. House of Representatives before the 2018 election?
    (c) What job or office does JD Vance hold?
    (d) What job or office does John Roberts hold?

    *Source: Inspired by ANES (American National Election Survey).*

The post-treatment questionnaire assessed affective polarization, political participation, and democratic attitudes, with affective polarization measured first, followed by political participation and democratic attitudes. Exact item wording is provided below.

1. **Affective Polarization:** We ask you to rate your feelings toward political groups using a 'feeling thermometer'. Ratings from 50–100 indicate warm/favorable feelings, 0–50 indicate cold/unfavorable feelings, and 50 is neutral.

   (a) How would you rate your feelings toward Republicans? *(slider: 0 = cold, 100 = warm)*

   (b) How would you rate your feelings toward Democrats? *(slider: 0 = cold, 100 = warm)*

   *Source: ANES (American National Election Study) and (1).*

2. **Democratic Attitudes:**

   (a) **SWD:** On the whole, how satisfied are you with how democracy works in the U.S.A? *(1 = Not at all satisfied, 11 = Very satisfied) European Values Survey:*

   (b) **Broken Political System** Please indicate your agreement: "The political system is broken." *(10-point scale: 1 = Strongly disagree, 11 = Strongly agree)*

   *Hope Not Hate 2024 Report*

3. **Political Participation:**

   (a) Please indicate whether you have engaged in any of the following during the past 12 months:
   - Taken part in a protest, march, or demonstration on a national or local issue.
   - Signed a petition (paper or online) about a political or social issue.
   - Posted a message on social media about a political issue.
   - Voted in the 2024 presidential election.

   *ANES (American National Election Study)*

***Indices.*** **Political sophistication** was measured using a composite index combining political interest and political knowledge. Political interest was assessed using a self-reported 10-point scale (1 = lowest interest, 10 = highest interest), and political knowledge was measured using a five-item scoring scale (range: 0–5), where there our five factual questions that are coded as correct or incorrect. Both measures were linearly rescaled to range from 0 to 1 using min–max normalization (political interest: $(x - 1)/9$; political knowledge: $(x - 0)/5$). The political sophistication index was computed as the unweighted mean of the two normalized measures and therefore ranged from 0 to 1, with higher values indicating greater political sophistication.

**Affective polarization** was measured using respondents' affective evaluations of the two major U.S. political parties. Participants reported their feelings toward Democrats and Republicans on separate feeling thermometer scales. Responses were converted to numeric values prior to analysis. Two measures of affective polarization were constructed. First, a signed difference score was calculated by subtracting respondents' affect toward Republicans from their affect toward Democrats, with positive values indicating more favorable affect toward Democrats and negative values indicating more favorable affect toward Republicans. Second, an absolute difference score was calculated as the absolute value of the difference between affect toward Democrats and affect toward Republicans, capturing the overall magnitude of affective polarization regardless of partisan direction. Higher values on the absolute measure indicate greater affective polarization.

**Appendix A2: Sampling and Participants.** We recruited $N = 1{,}021$ U.S.-based participants via Prolific in July 2025 using the platform's U.S.-representative sampling framework, which approximates population distributions for age, gender, ethnicity, and party identification. Additional details on Prolific's representative sampling methodology are available at https://researcher-help.prolific.com/en/articles/445161-what-are-representative-samples-on-prolific.

We focus on the United States as our case of analysis because much of the foundational empirical literature on emotion and political behavior has been developed in the U.S. context, allowing our results to speak directly to existing theory while providing a clear baseline for future comparative research.

A total of 29 respondents were excluded for failing to meet pre-registered inclusion criteria, including (1) passing attention checks, (2) demonstrating adequate comprehension, and (3) providing valid body-mapping responses. The final analytic sample therefore consists of 992 participants. Figure S1 presents descriptive statistics for this sample.

The Prolific U.S.-representative sampling procedure yielded a sample that closely matches key demographic characteristics of the U.S. population. The final analytic sample exhibits an approximately even gender balance, as well as age and ethnic distributions that align with benchmarks from U.S. Census data. Partisan identification is likewise broadly representative, including Democrats, Republicans, and a substantial share of respondents who do not affiliate with either major party, consistent with recent survey evidence documenting a historically high proportion of political independents in the United States (2). With respect to educational attainment, the sample is slightly more educated than national benchmarks from U.S. Census

data. Finally, as is typical of non-probability samples drawn from online panels, participants in the study tend to be relatively politically sophisticated, a consideration we account for when interpreting the results.

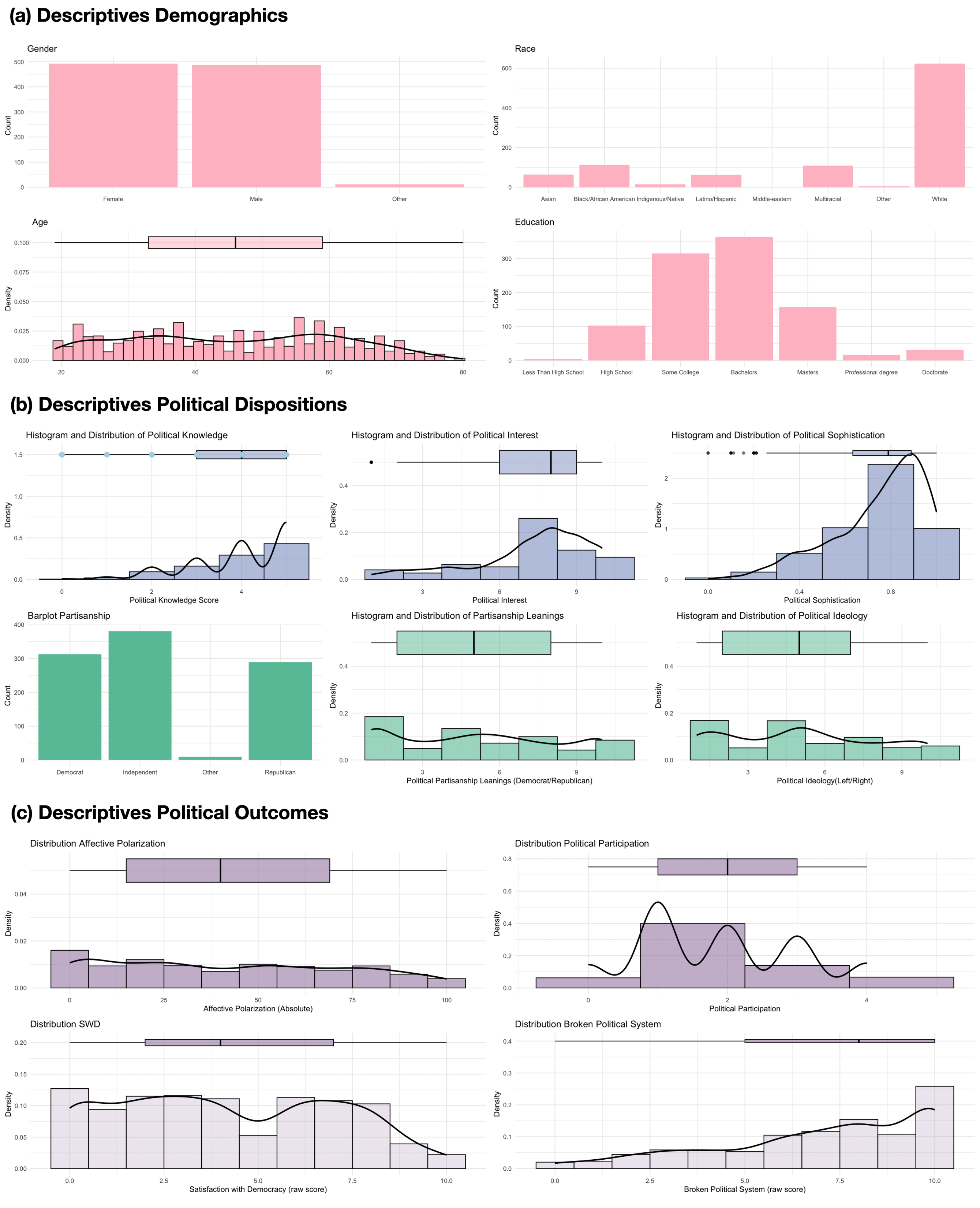


**Fig. S1.** Descriptives of Participants (N = 992).

**Appendix A3: emBODY-tool Protocol.** The experiment, including the emBODY-tool protocols, was implemented on the Gorilla platform. The non-political and political protocols are publicly available at the links below and may be viewed, tested, and duplicated for use in other research projects.

- Gorilla link: emBODY-tool non-political protocol - as done in (3) and (4).
- Gorilla link: emBODY-tool political protocol

As described in the method section, the political emBODY protocol consists of an emBODY task consisting of three subtasks: (1) Issue Selection: Participants will choose a political issue that elicits the target emotion (anger, disgust, anxiety, depression, or hope) from a drop-down menu. The list of political issues is based on Pew Research's ranking of key public concerns among US citizens (5) and is available in Figure S2. (2) Emotion Rating: Participants will rate the intensity of the emotion chosen for that issue on a scale from 0 to 100. (3) Bodily Sensation Mapping: Using the emBODY tool, participants will indicate how the emotion regarding their selected political issue affects their bodily sensations, with activation regions marked in red and deactivation regions in blue.

In Figure S2, we summarize the descriptives of the political issue task. Panel (a) displays the percentage of respondents who selected each political issue as a source of particular political emotions, showing that issues such as the state of the political system and the economy elicit especially strong emotional responses across multiple emotions. Panel (b) presents the distribution of self-reported emotional intensity by issue, indicating that most emotions—such as anger, anxiety, depression, and disgust—are heavily skewed toward higher levels of reported intensity (e.g., respondents frequently reporting feeling very angry). The notable exception is hope, where reported emotional intensity is generally lower and more evenly distributed. Panel (c) compares partisan differences in issue selection within each emotional category, revealing that Democrats and Republicans often associate different issues with similar emotions, reflecting distinct emotional and issue-based polarization patterns.

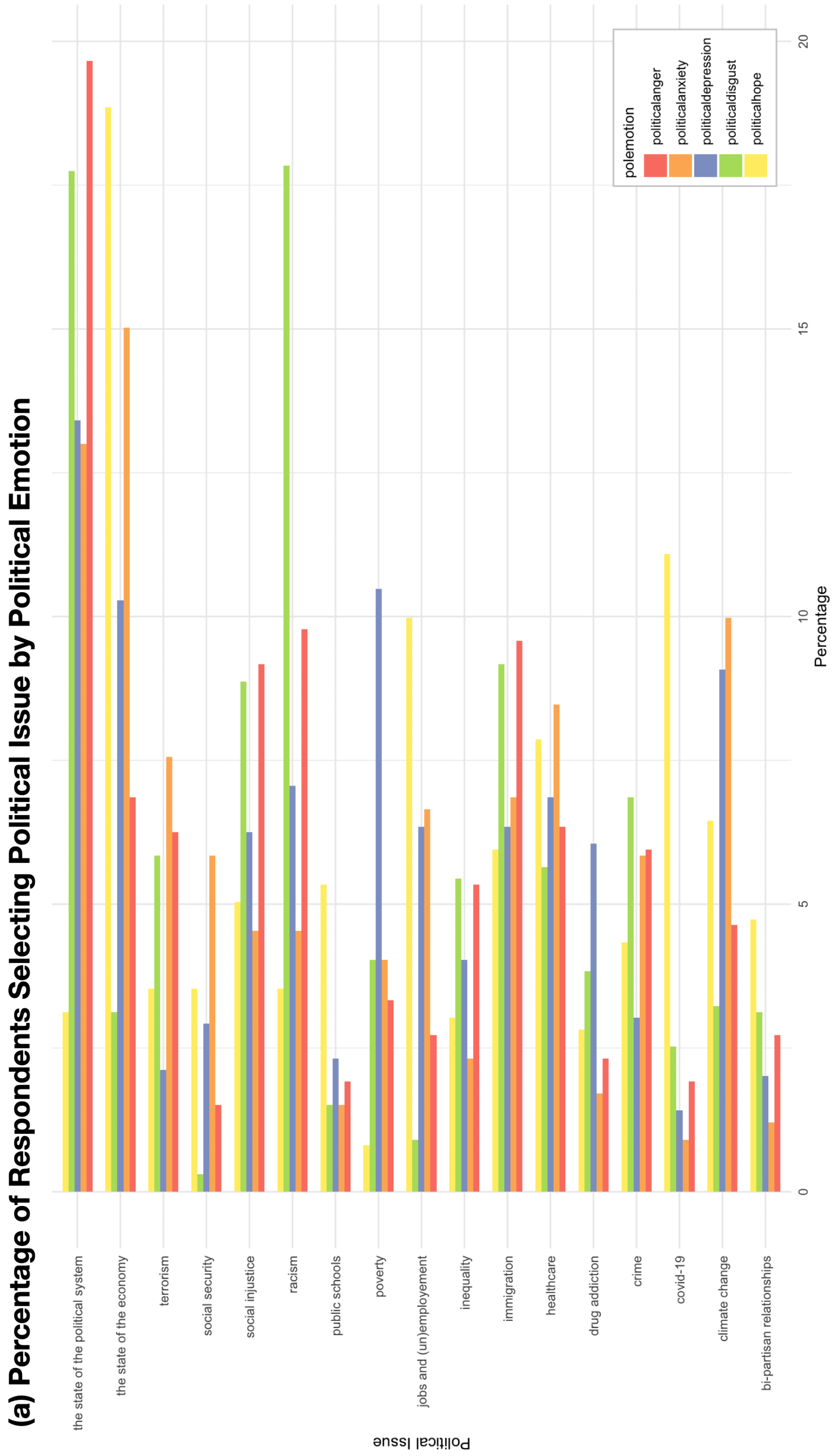


**Fig. S2. A closer look at political emotions.**

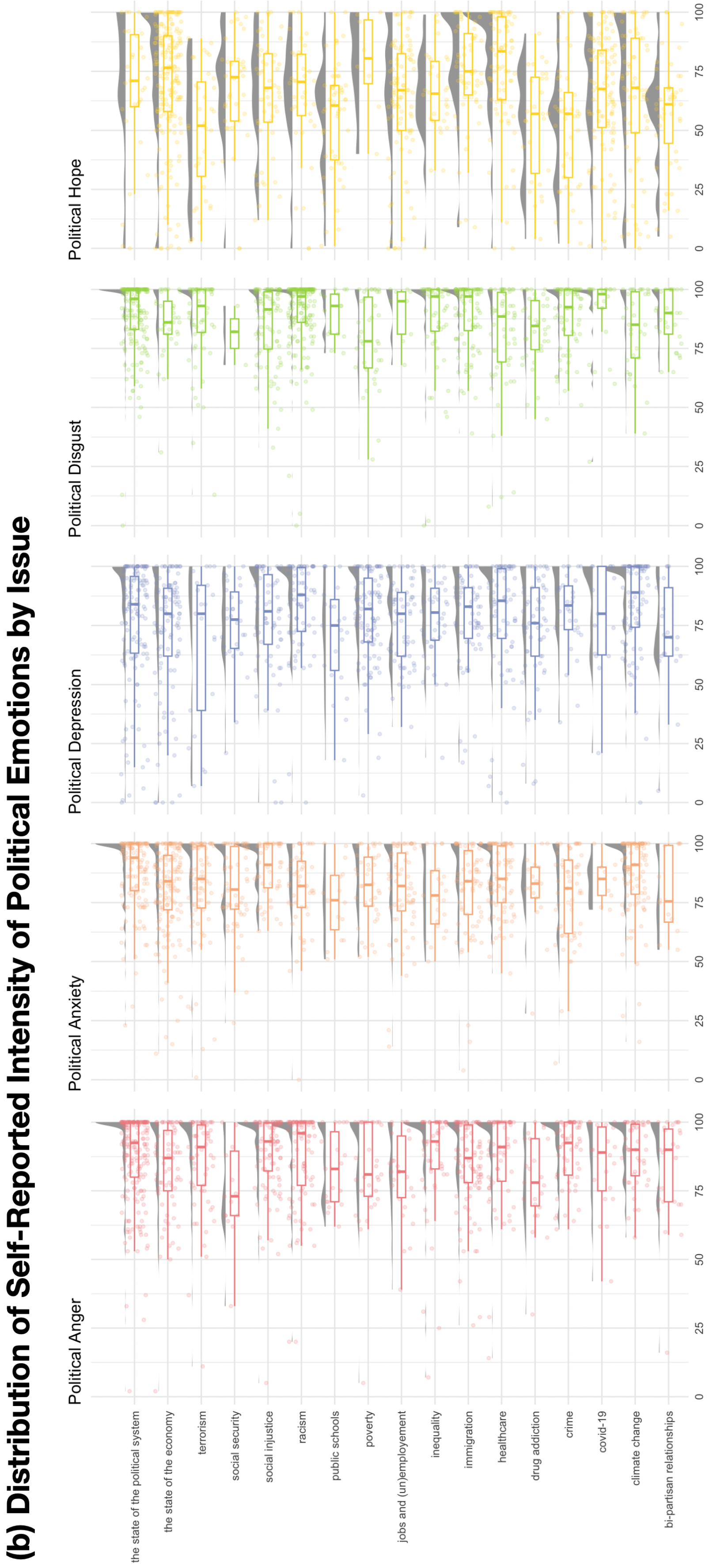


**Fig. S2.** Figure S2 (continued)

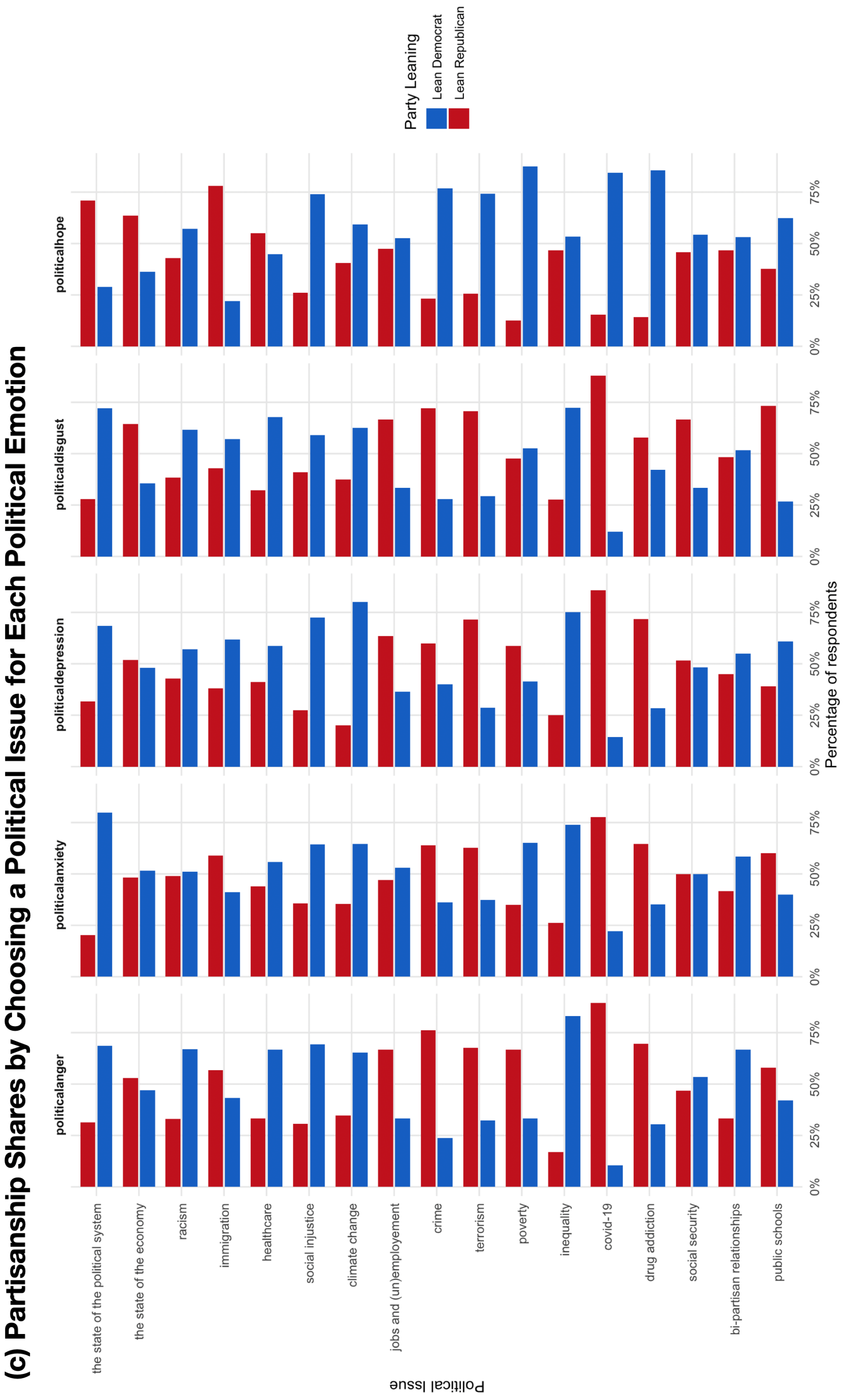


**Fig. S2.** Figure S2 (continued)

**Appendix A4: Embodied Impact: Conceptualization and Correlation with Self-reported Emotional Intensity for Political Emotions.** From the experimental protocols, we derive multiple metrics to form a composite measure of embodied impact, summarized in Table S1.

**Table S1. Bodily Sensation Mapping Metric: Embodied Impact**

| **Embodied Impact** | The embodied impact of an emotional response. Embodied Impact is an index of three embodied metrics that measure the breadth and depth of the embodied emotional response: size, intensity, and spread. | |
|---|---|---|
| **Metric** | **Formula** | **Description** |
| **Size (i.e., the proportion of painted pixels)** | $\frac{\lvert\{p \in M : p_{value} \neq 0\}\rvert}{\lvert\{p \in M\}\rvert}$ | The proportion of the body that is colored (6–8). Specifically, the number of non-zero pixels (i.e., painted pixels) was divided by the total number of pixels for each participant for each emotion. This measurement does not consider the intensity value of a pixel but only whether it was colored (1) or not (0). The size score ranges from 0 to 1, where 1 means the entire body was colored by the participant. |
| **Intensity (i.e., intensity of painted pixels)** | $\frac{\sum_{p \in M, p_{value} \neq 0} p_{value}}{\lvert\{p \in M : p_{value} \neq 0\}\rvert \times 5}$ | Intensity is calculated as the ratio of the sum of the intensity of the painted pixels to the maximum possible intensity. This provides a normalized measure of intensity, where 1 indicates the highest possible intensity (i.e., all painted pixels are at their maximum value of 5), and values less than 1 indicate lower intensity. NAs were assigned if no pixels were painted. |
| **Spread (i.e., ROI coverage)** | $\frac{\lvert\{R_i \in R : \exists p \in R_i, p_{value} \neq 0\}\rvert}{\lvert R \rvert}$ | The body is divided into seven Regions of Interest (ROIs): head and neck, chest, abdomen, legs, arms, hands, and feet. ROI coverage refers to how many body parts contain painted pixels. If the entire body is covered, this receives a score of 1; if only the head is colored, this receives a score of 1/7 = 0.14. |

Figure S3 displays the relationship between embodied impact (x-axis) and self-reported emotional intensity (y-axis) across five political emotions: anger, anxiety, depression, disgust, and hope. Each panel includes a fitted regression line with 95% confidence intervals and the corresponding correlation coefficient (r). Across all emotions, the relationships are weak and near zero, indicating little to no systematic association between embodied impact and the intensity with which respondents reported feeling each political emotion. Emotional intensity tends to cluster at high levels for most emotions, which is not the case for embodied impact.

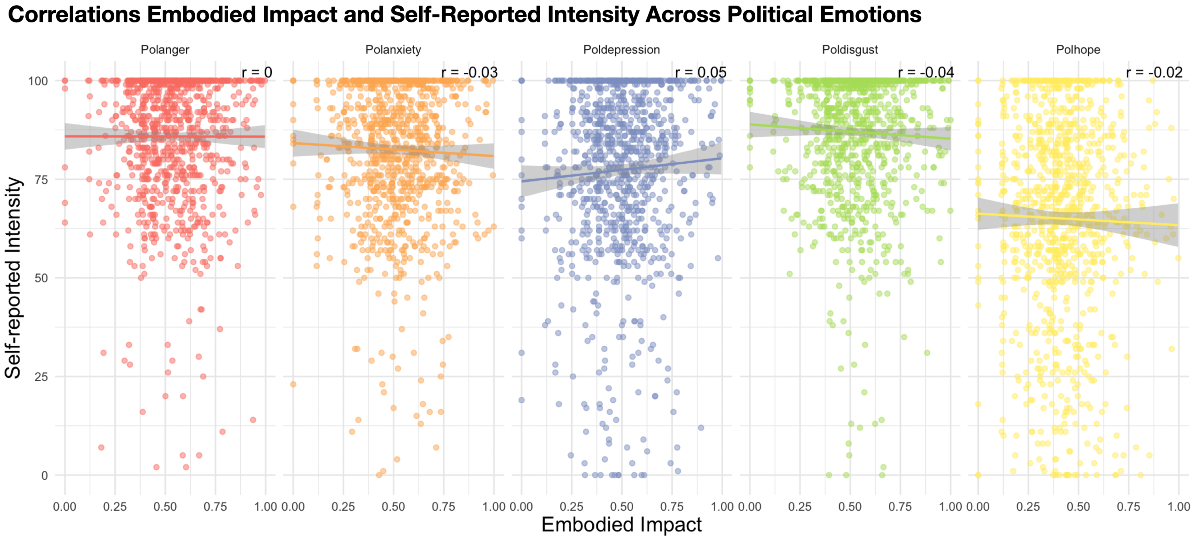


**Fig. S3.** Correlations Between Embodied Impact and Self-Reported Emotional Intensity

**Appendix A5: Word Cloud and Sentiment Analysis of the Open Question on the emBODY tool.** The word cloud in Figure S4 summarizes participants' open-ended reflections on completing the emBODY-tool. A surprising number of participants chose to provide written reflections in this section, suggesting that many found the task engaging and distinct from typical survey experiences. The most frequent words—such as interesting, feel, body, and emotions—indicate that respondents actively engaged with the exercise and found it meaningful. Positive descriptors (e.g., interesting, easy, engaging) appeared more often than negative ones (e.g., difficult, confusing, hard), though both were common. This pattern supports the validity of the tool, as it suggests that participants were genuinely reflecting on the embodied aspects of their emotional experiences. The variation in responses—some describing the task as easy or fun, others as challenging or difficult—further underscores individual differences in people's ability to access and articulate bodily feelings associated with emotions.

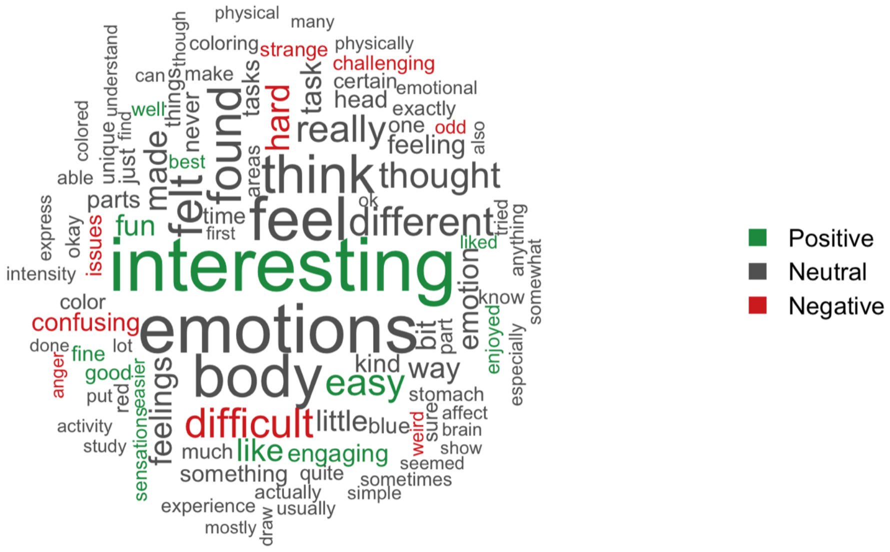


N=992 | Responded: 966 (97.4%)
Any positive (lexicon): 574 (57.9% of all; 59.4% of respondents)
Any negative (lexicon): 384 (38.7% of all; 39.8% of respondents) | Both: 148

**Fig. S4.** Wordcloud Open-Ended emBODY-tool Question

## Appendix B: Supplementary Analyses

In this section, we provide all supplementary analyses, structured by research question.

**Appendix B.1: RQ1.** To assess the robustness of our findings, we conducted several complementary analyses. First, we run equivalance testing on the results from out main repeated measures ANOVA. Second, we present the preregistered aggregated analysis without control variables (see Figure S5). Third, we ran separate analyses for activation and deactivation maps, as specified in the preregistration (see Figure S6). Finally, as preregistered we conducted a cosine similarity assessment as part of the topographical analysis (see Figure S7). Cosine similarity captures both the pattern and relative intensity of painted activations by comparing the angle between two high-dimensional vectors, yielding values between 1 and 1 (see (9)). Values closer to 1 indicate highly similar activation patterns, values near 0 indicate orthogonal (unrelated) patterns, and values closer to 1 indicate opposing patterns of activation and deactivation. Importantly, cosine similarity preserves the directionality of activation and deactivation, rather than collapsing effects to magnitude alone.

To complement the repeated-measures ANOVA results reported in the main text, and as preregistered, we conducted equivalence tests using the two one-sided tests (TOST) procedure (Lakens, 2017) to compare each political emotion to its non-political counterpart on embodied-impact scores. A Cohen's $d$ equivalence margin of $\pm 0.25$ was specified. To translate this standardized bound to the raw scale of the dependent variable, we multiplied $\pm 0.25$ by the pooled observed standard deviation of the two relevant emotion conditions. Each model-based estimated marginal mean contrast was then tested against these emotion-specific raw equivalence bounds using the contrast's standard error and degrees of freedom returned by the covariate-adjusted model. We report Benjamini–Hochberg adjusted $p$-values for the conventional null hypothesis significance test (NHST) and the corresponding TOST $p$-values, classifying results as (a) significant and not equivalent, (b) non-significant but equivalent, or (c) inconclusive.

**Table S2. Two one-sided test (TOST) results comparing political and non-political emotions on embodied-impact scores (Cohen's $d$ margin ±0.25). Raw bounds correspond to the pooled observed standard deviation of each emotion pair. Model-based contrasts include covariate adjustment for participant characteristics.**

| Emotion pair | Estimate | SE | df | Raw bounds | $p_{\text{BH}}$ | $p_{\text{TOST}}$ | Decision |
|---|---|---|---|---|---|---|---|
| Anger – Polanger | 0.009 | 0.006 | 975 | [0.0446, 0.0446] | .180 | $8.6 \times 10^{-9}$ | n.s. (BH) + Equivalent |
| Anxiety – Polanxiety | 0.039 | 0.006 | 975 | [0.0441, 0.0441] | $1.6 \times 10^{-11}$ | .191 | sig (BH) + Not equivalent |
| Depression – Poldepression | 0.053 | 0.007 | 975 | [0.0483, 0.0483] | $1.6 \times 10^{-13}$ | .730 | sig (BH) + Not equivalent |
| Disgust – Poldisgust | 0.055 | 0.006 | 975 | [0.0420, 0.0420] | $5.5 \times 10^{-20}$ | .988 | sig (BH) + Not equivalent |
| Hope – Polhope | 0.045 | 0.006 | 975 | [0.0431, 0.0431] | $1.2 \times 10^{-12}$ | .606 | sig (BH) + Not equivalent |

Among the five emotion pairs, only *Anger–Polanger* met the criterion for statistical equivalence within the preregistered margin ($p_{\text{TOST}} < .05$), indicating no practically meaningful difference in embodied impact between political and non-political anger after adjusting for covariates. For all other emotions, the TOST procedure did not reject the null of non-equivalence, consistent with meaningful political–non-political differences in embodied responses.

**RQ1 Aggregated Analysis: ANOVAs Emotions and Embodied Impact (no controls).**

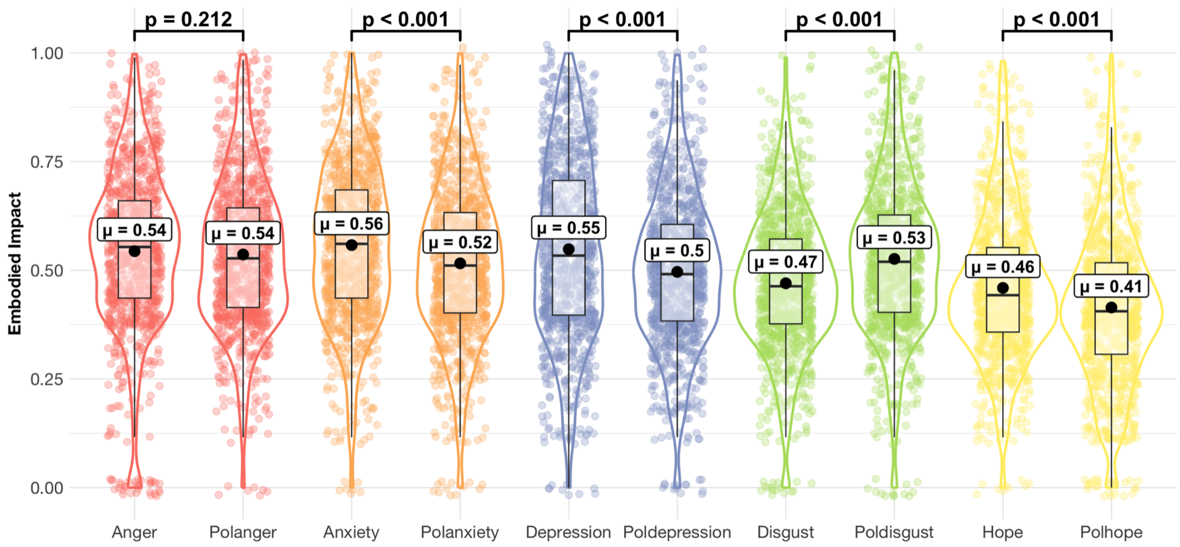


**Fig. S5.** Violin plots show the distribution of embodied impact scores (0–1) for each emotion, separated by political (right) and non-political (left) contexts. Black dots mark the mean ($\mu$), and boxplots indicate the interquartile range. Statistical comparisons between political and non-political versions of each emotion are shown above each pair. For all emotion pairs except anger, political and non-political emotions differ significantly in embodied impact ($p < .001$). This is done on 992 respondents, no control variables included.

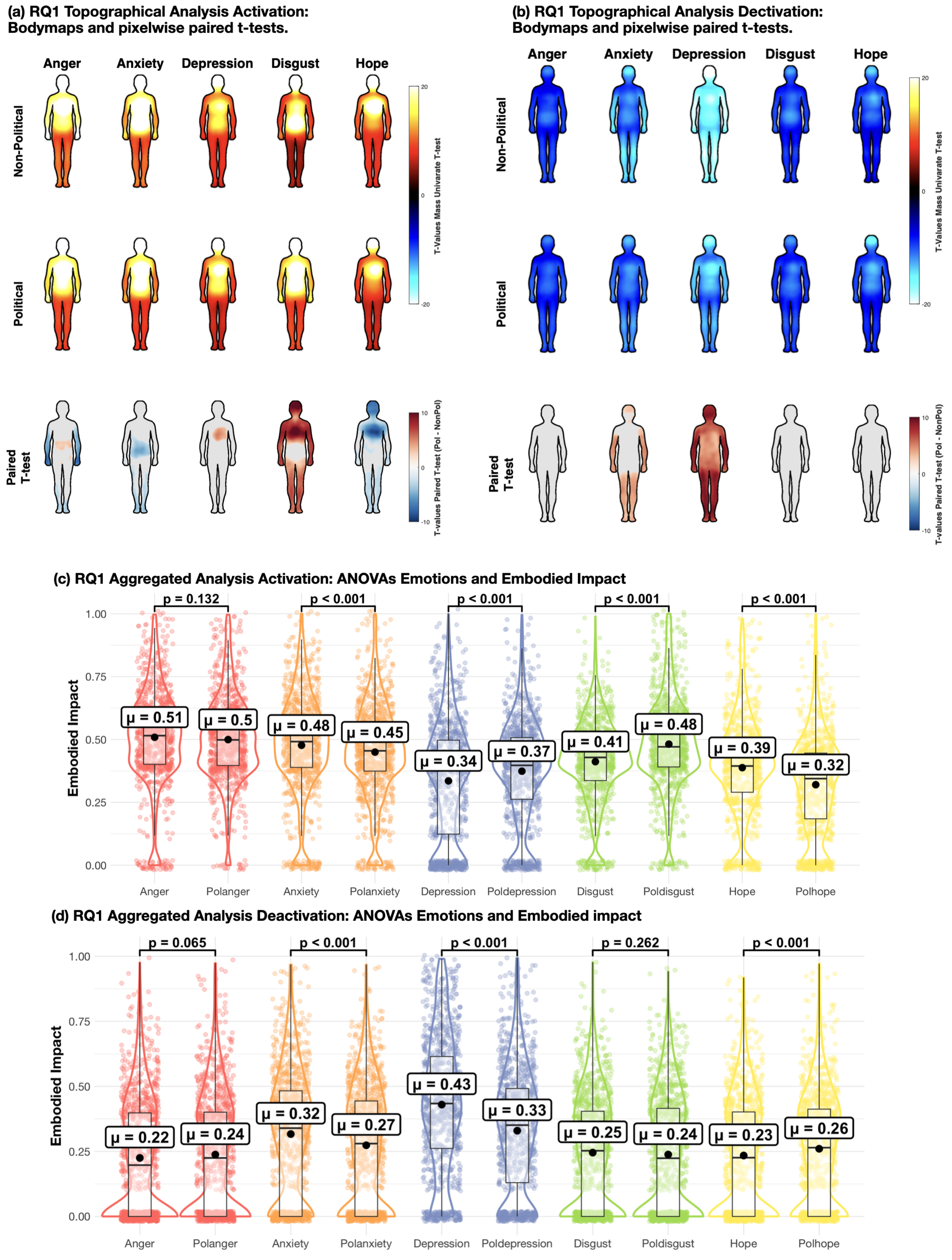


**Fig. S6.** Panels (a) and (b) show topographical analyses of bodily activation (warm colors) and deactivation (cool colors) for each emotion in political and non-political contexts, along with pixelwise paired t-tests comparing the two. Panels (c) and (d) present aggregated analyses of embodied impact derived from these maps, showing mean differences and statistical comparisons across emotions.

# RQ1 Cosine Similarity Matrix

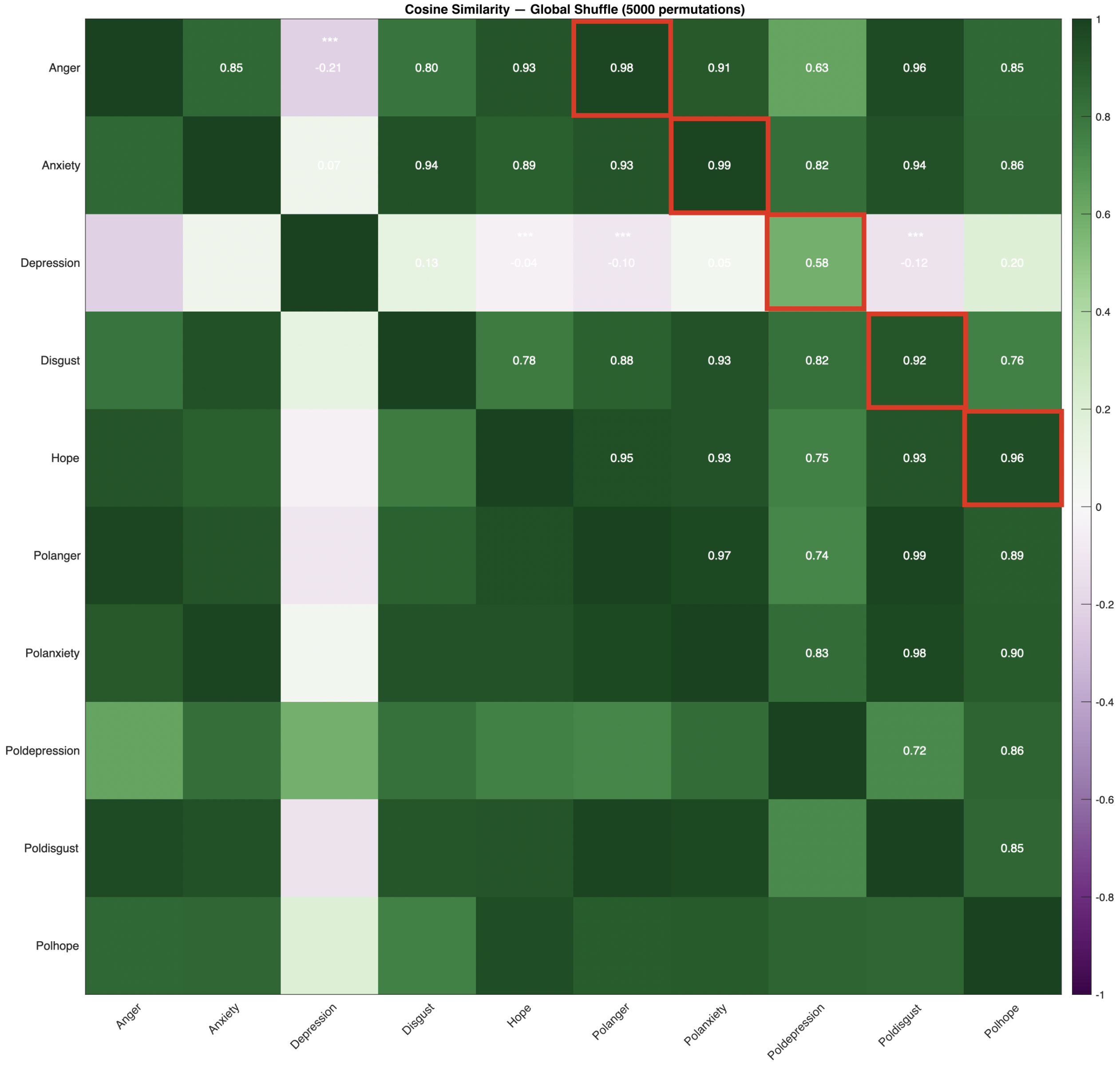


**Fig. S7.** Cosine Similarity Matrix: This matrix displays pairwise cosine similarity scores between average embodied emotion maps. Higher values (darker green) indicate greater spatial similarity in bodily activation patterns across emotions. Red rectangles highlight political and non-political counterparts (e.g., anger vs. polanger). The strong diagonal and high off-diagonal similarities suggest that political and non-political versions of each emotion share broadly similar embodied representations. However, some pairs—particularly depression and political depression—show comparatively lower similarity, indicating subtle differences in how these emotions are experienced bodily in political versus non-political contexts.

**Appendix B.2: RQ2.** To further assess the robustness of our findings, we conducted several complementary analyses. First, we conduct TOST equivalence testing on our main model. Second, repeated the aggregated analysis without including control variables (see Figure S8). Third, we replicated the aggregated analysis using self-reported emotional intensity as the outcome measure, which we did not preregister (see Figure S9). Fourth, we reran the analysis focusing exclusively on non-political emotions (see Figure S10). Fifth, as preregistered we performed a pixelwise Spearman correlation using continuous measures of political sophistication and political partisanship (see Figure S11). Sixth, we conducted separate analyses for activation and deactivation maps, as specified in the preregistration (see Figure S12 and S13). And finally, for political sophistication, we conducted the analysis of the main finding seperately by political interest and political knowledge (see Figure S14), and for partisanship, we replicated the main analysis of both the political and non-political emotional conditions on ideological left/right self-placement (see Figure S15).

To evaluate whether statistically non-significant effects could be interpreted as evidence for the absence of meaningful group differences, we conducted equivalence tests (TOST; 10) for all pairwise contrasts within each emotion condition. As preregistered, the equivalence margin was set at Cohen's $d = \pm 0.25$.

For both political sophistication and partisanship, the standardized margin was converted to the raw scale separately for each emotion as $\pm 0.25 \times SD_{\text{emotion}}$, using the observed standard deviation of *impact* within that emotion category. Each estimated marginal mean (EMM) contrast from the mixed ANOVA models (which included demographic controls) was then tested against these emotion-specific bounds using two one-sided $t$-tests with the model-derived standard errors and degrees of freedom.

We report both the Benjamini–Hochberg adjusted NHST $p$-values and the TOST $p$-values. Results are classified as (a) *significant and not equivalent* (i.e., non-trivial differences), (b) *non-significant but equivalent* (i.e., trivially small effects), or (c) *inconclusive* when neither hypothesis can be rejected.

**Table S3. Equivalence tests (TOST) for political sophistication across political emotion conditions. Equivalence bounds were set at Cohen's $d = \pm 0.25$, converted to raw units using the observed standard deviation of *impact* within each emotion. Reported are estimated marginal mean differences (High–Low sophistication), standard errors, Benjamini–Hochberg adjusted $p$-values for NHST, and TOST results.**

| Emotion | Estimate | SE | df | $SD_{\text{obs}}$ | $p_{\text{BH}}$ | $p_{\text{TOST}}$ | Equivalence | Decision |
|---|---|---|---|---|---|---|---|---|
| Polanger | 0.026 | 0.012 | 910 | 0.170 | .053 | .082 | Not equivalent | Inconclusive |
| Polanxiety | 0.018 | 0.012 | 910 | 0.175 | .127 | .018 | Equivalent ($\lvert d\rvert \leq .25$) | n.s. + Equivalent |
| Poldepression | 0.022 | 0.012 | 910 | 0.179 | .090 | .038 | Equivalent ($\lvert d\rvert \leq .25$) | n.s. + Equivalent |
| Poldisgust | 0.032 | 0.012 | 910 | 0.176 | .046 | .167 | Not equivalent | sig + Not equivalent |
| Polhope | 0.027 | 0.012 | 910 | 0.178 | .053 | .075 | Not equivalent | Inconclusive |

**Table S4. Equivalence tests (TOST) for partisanship across political emotion conditions. Equivalence bounds were set at Cohen's $d = \pm 0.25$, converted to raw units using the observed standard deviation of *impact* within each emotion. Reported are estimated marginal mean differences (Lean Democrat–Lean Republican), standard errors, Benjamini–Hochberg adjusted $p$-values for NHST, and TOST results.**

| Emotion | Estimate | SE | df | $SD_{\text{obs}}$ | $p_{\text{BH}}$ | $p_{\text{TOST}}$ | Equivalence | Decision |
|---|---|---|---|---|---|---|---|---|
| Polanger | 0.033 | 0.011 | 910 | 0.170 | .004 | .192 | Not equivalent | sig (BH) + Not equivalent |
| Polanxiety | 0.038 | 0.011 | 910 | 0.175 | .001 | .315 | Not equivalent | sig (BH) + Not equivalent |
| Poldepression | 0.049 | 0.012 | 910 | 0.179 | $< .001$ | .651 | Not equivalent | sig (BH) + Not equivalent |
| Poldisgust | 0.039 | 0.012 | 910 | 0.176 | .001 | .346 | Not equivalent | sig (BH) + Not equivalent |
| Polhope | -0.015 | 0.012 | 910 | 0.178 | .199 | .007 | Equivalent ($\lvert d\rvert \leq .25$) | n.s. (BH) + Equivalent |

Across both models, the equivalence testing revealed distinct patterns. For *political sophistication*, differences across sophistication levels were generally small and non-significant, with only *disgust* showing a statistically reliable and non-equivalent difference. In contrast, for *partisanship*, significant and non-trivial effects emerged for four of the five emotions—*anger*, *anxiety*, *depression*, and *disgust*, indicating consistent partisan differences in embodied impact. The effect for *hope*, however, was statistically equivalent to zero, suggesting minimal partisan variation for that emotion.

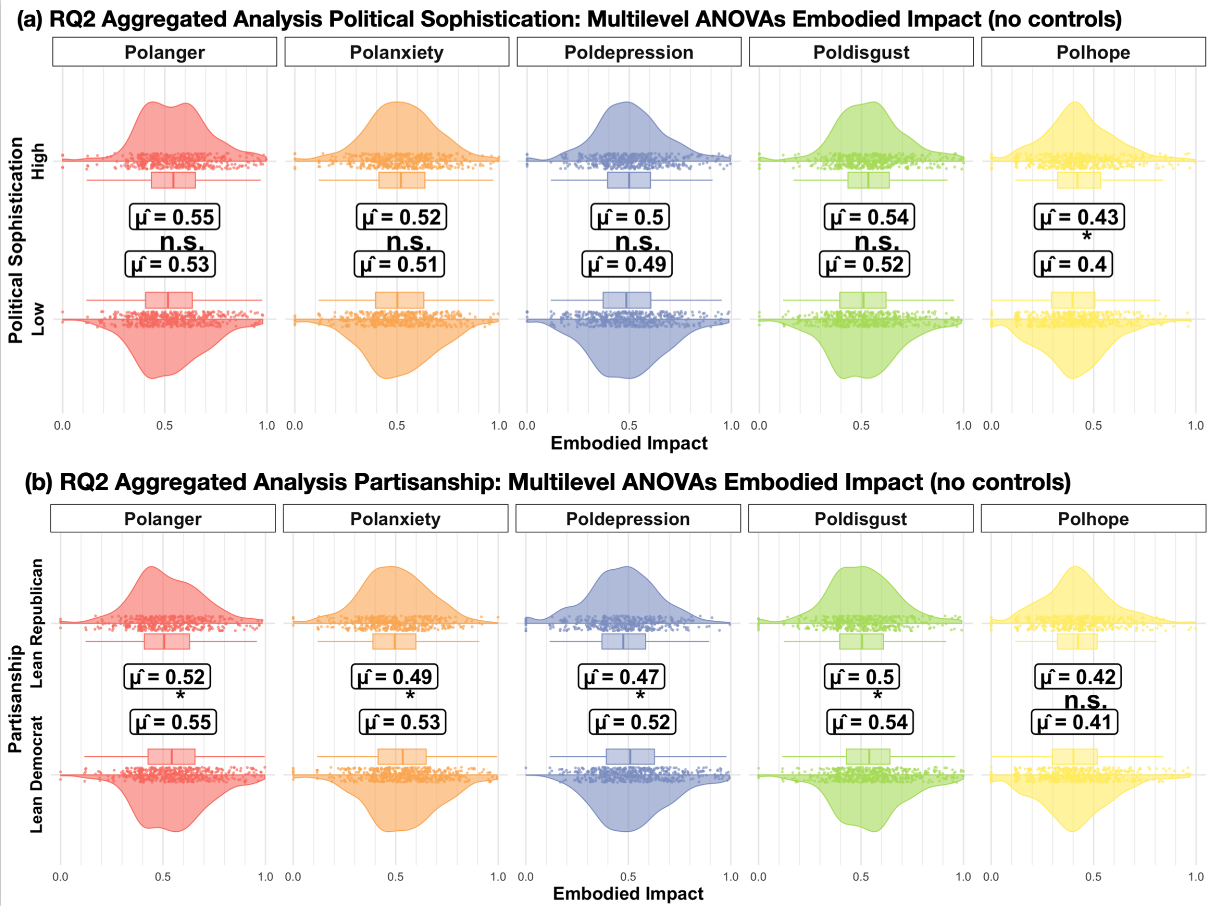


**Fig. S8.** Panel (a) shows embodied impact scores across levels of political sophistication (high vs. low), and Panel (b) displays the same analysis for political partisanship (leaning republican vs. leaning democrat). Mean estimates () are shown within each group. For political sophistication, differences were minimal and non-significant. In contrast, for political partisanship, Democrat-leaning participants exhibited greater embodied impact across all negative political emotions (anger, anxiety, depression, disgust), while political hope showed no significant difference between groups.

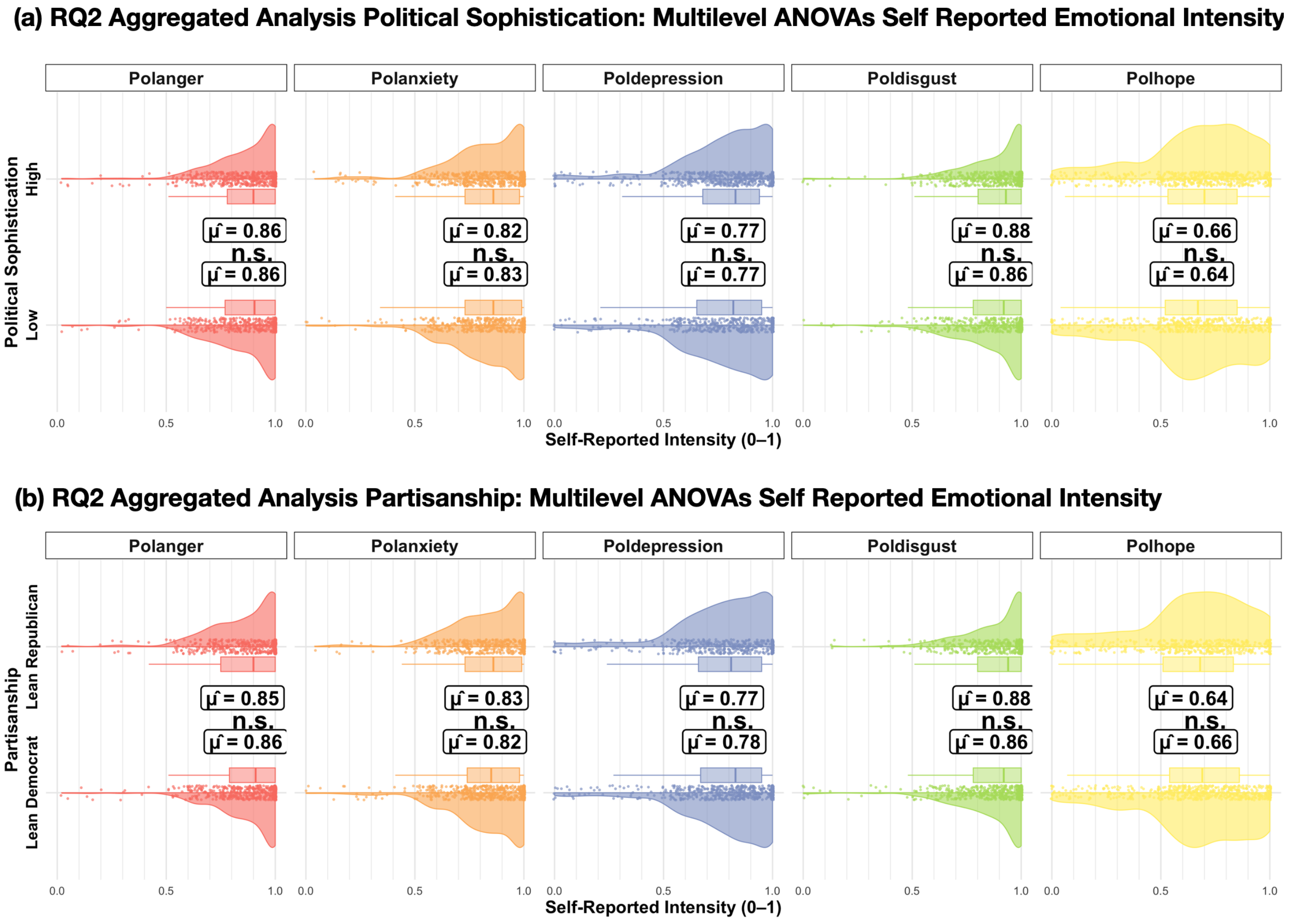


**Fig. S9.** Panel (a) shows self-reported emotional intensity scores across levels of political sophistication (high vs. low), and Panel (b) displays the same analysis for political partisanship (leaning republican vs. leaning democrat). Mean estimates () are shown within each group. There are no differences found between groups for any political emotion on self-reported emotional intensity.

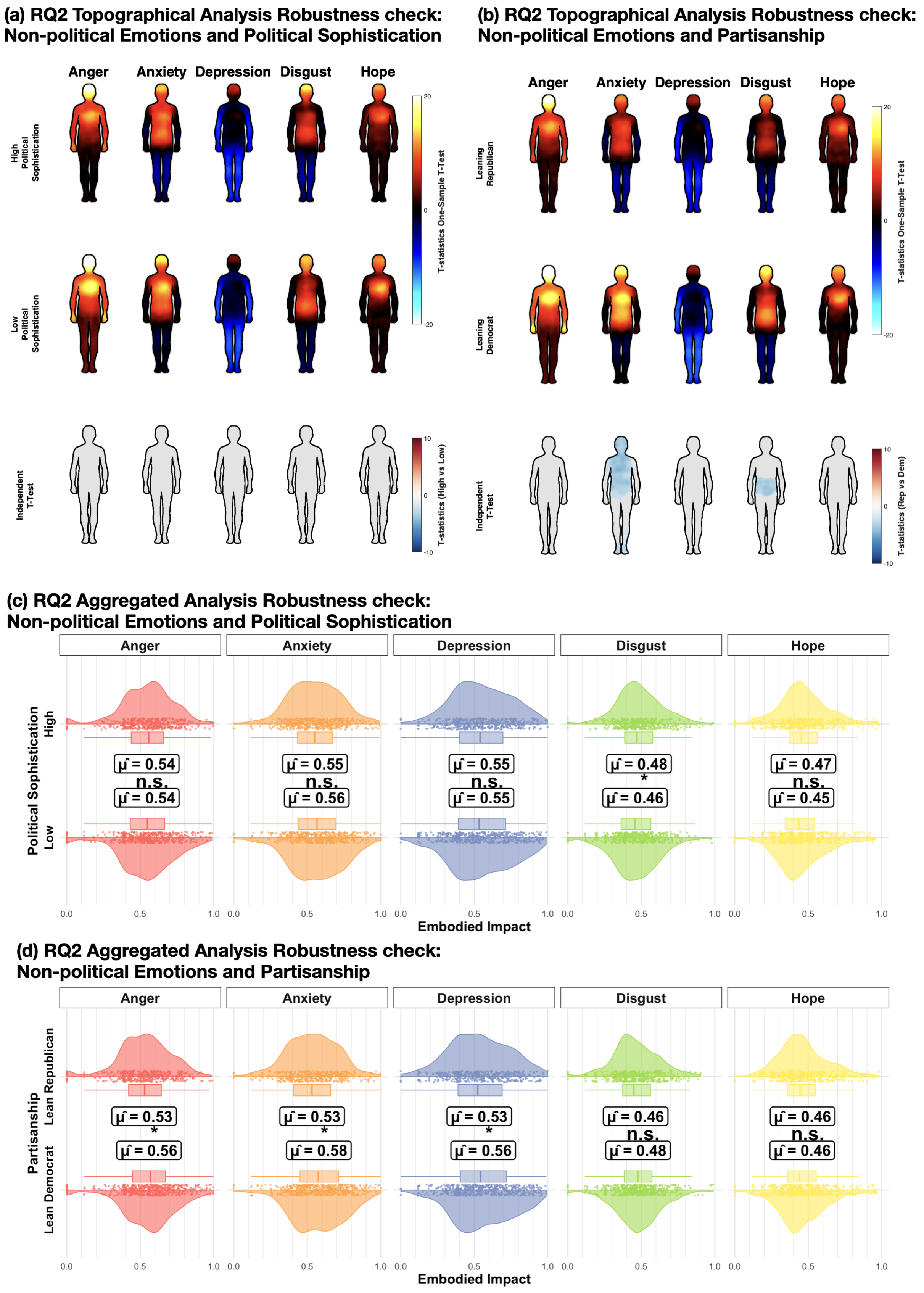


**Fig. S10.** Main Analysis Repeated for Non-Political Emotions (not preregistered).

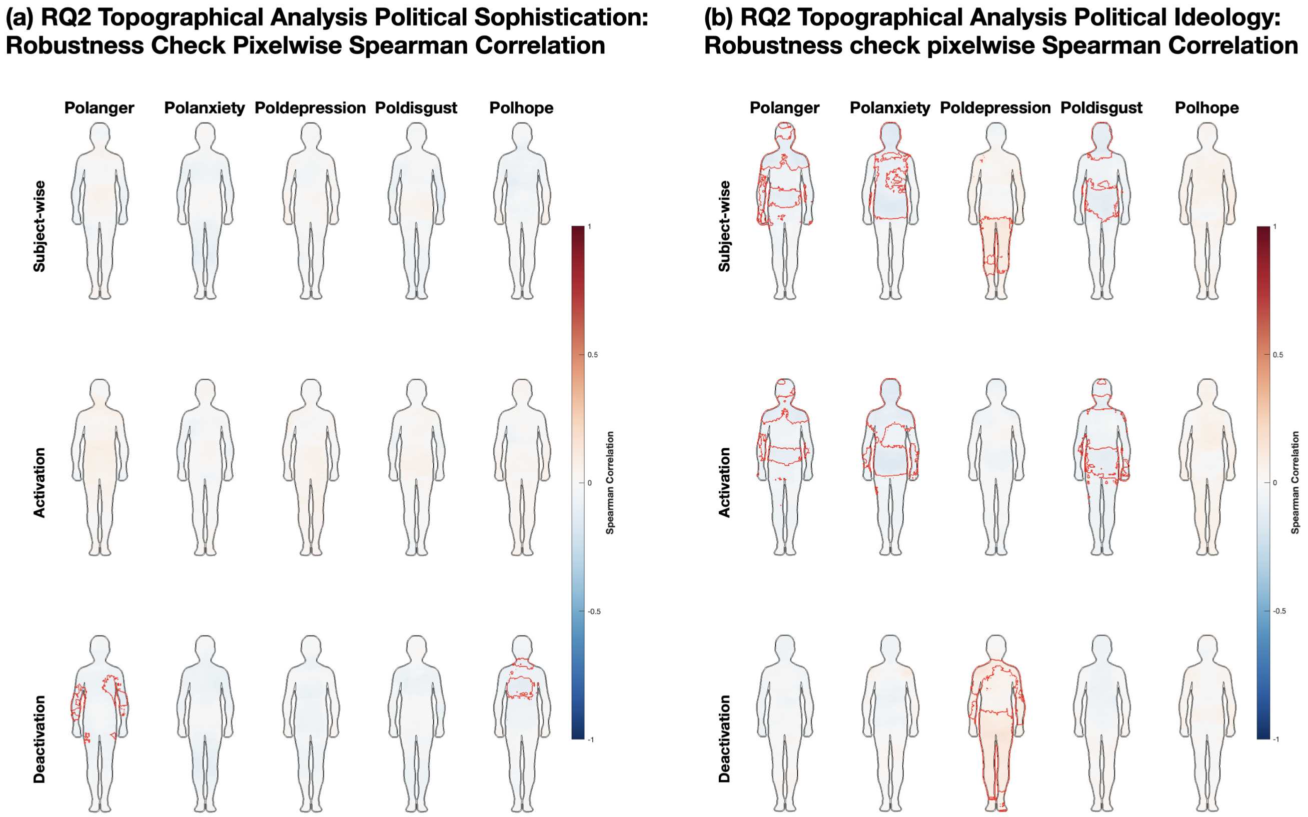


**Fig. S11.** Pixelwise Spearman correlations between embodied impact and political sophistication (continuous) (a) and political partisanship (continuous) (b). Each map shows the spatial Spearman correlations between embodied impact and the respective variable for political emotions, displayed separately for subject-wise, activation, and deactivation maps. Warmer colors indicate positive correlations and cooler colors indicate negative correlations. Statistically significant regions are outlined in red. Panel (a) shows few localized associations with political sophistication, whereas Panel (b) reveals broader significant relationships with political partisanship across several body regions.

## RQ2 Political Sophistication Robustness Check: Activation and Deactivation Maps

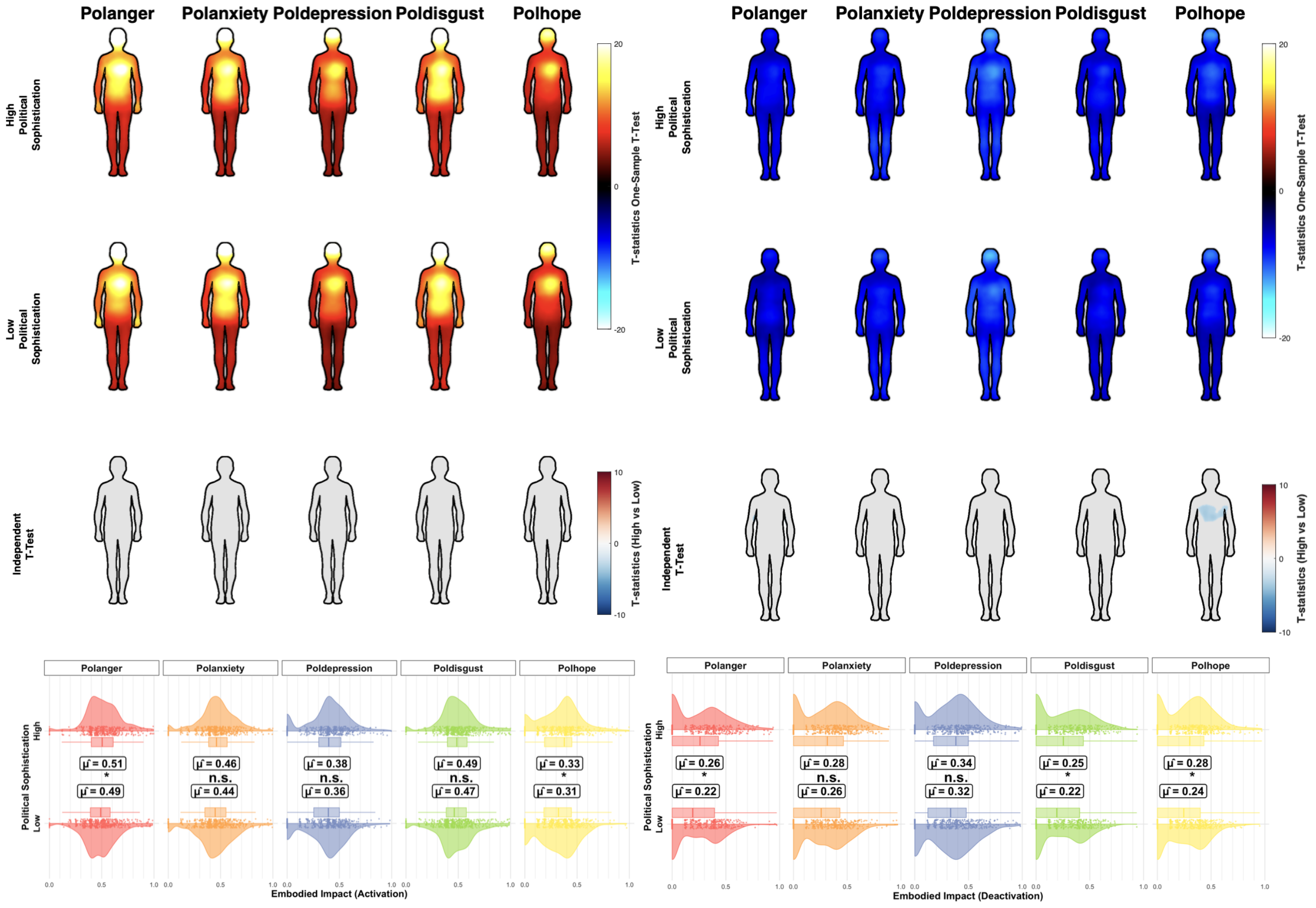


**Fig. S12.** RQ2 Political Sophistication, Main Analysis Repeated for Activations and Deactivation Maps Separately.

## RQ2 Partisanship Robustness Check: Activation and Deactivation Maps

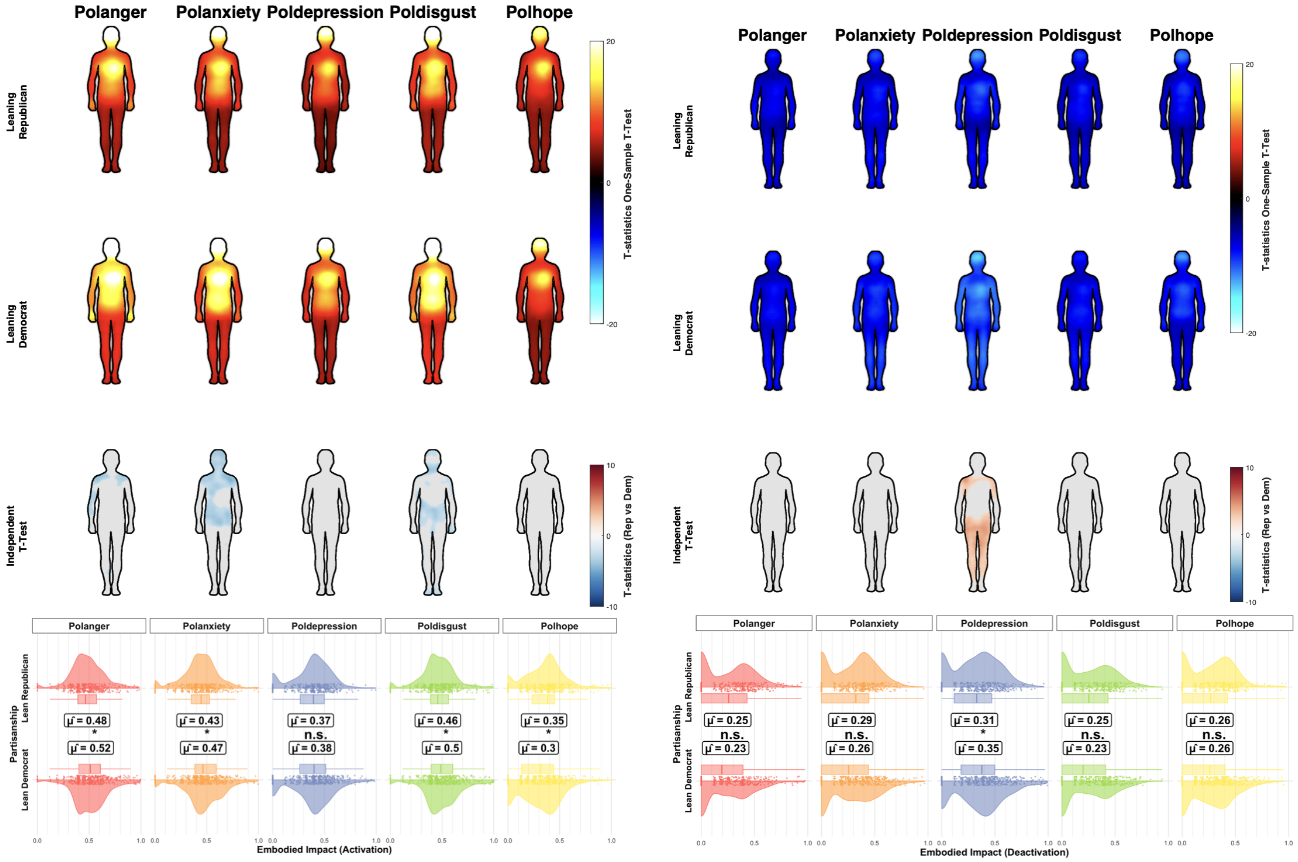


**Fig. S13.** RQ2 Partisanship: Main Analysis Repeated for Activations and Deactivation Maps Separately.

**Robustness Check RQ2 Political Sophistication: Separate Effects of Political Interest and Political Knowledge**

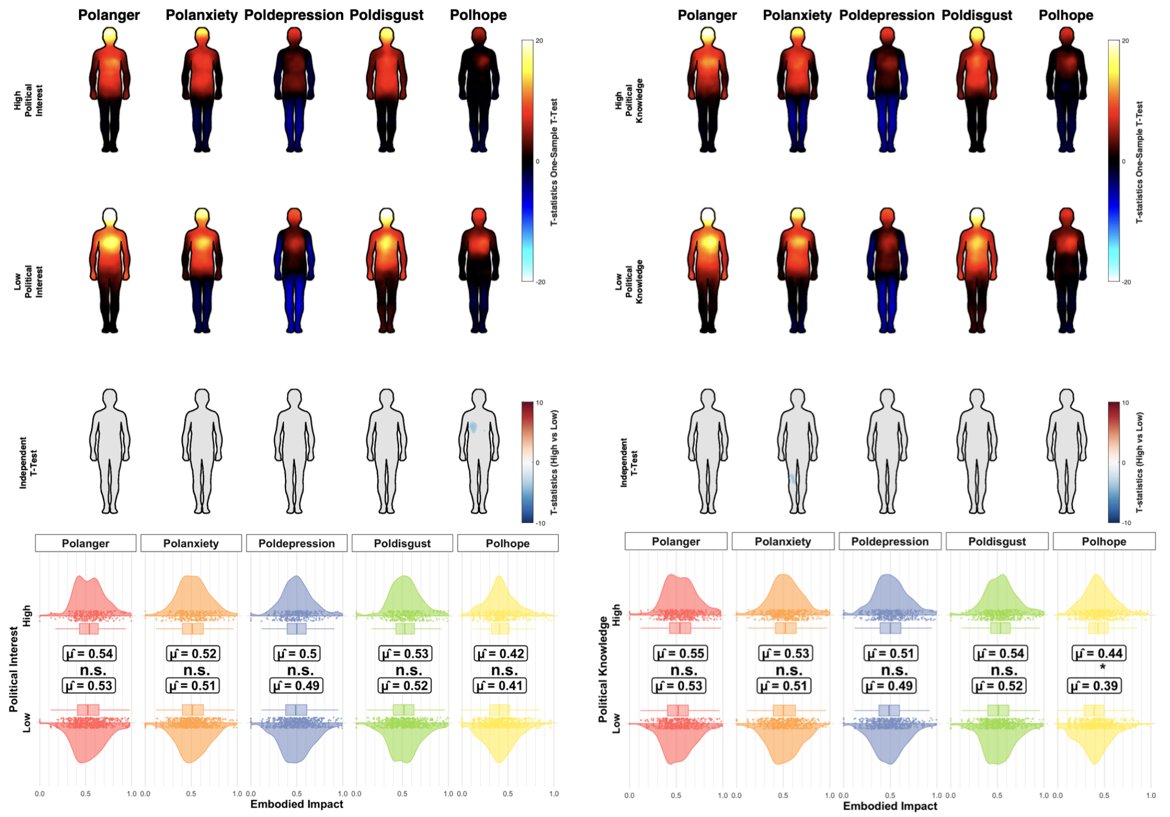


**Fig. S14.** Main Analysis of Political Sophistication Repeated for Political Interest and Political Knowledge Seperately Separately (not preregistered).

## Robustness check RQ2: Ideology (Left–Right Placement) as an Alternative to Partisanship

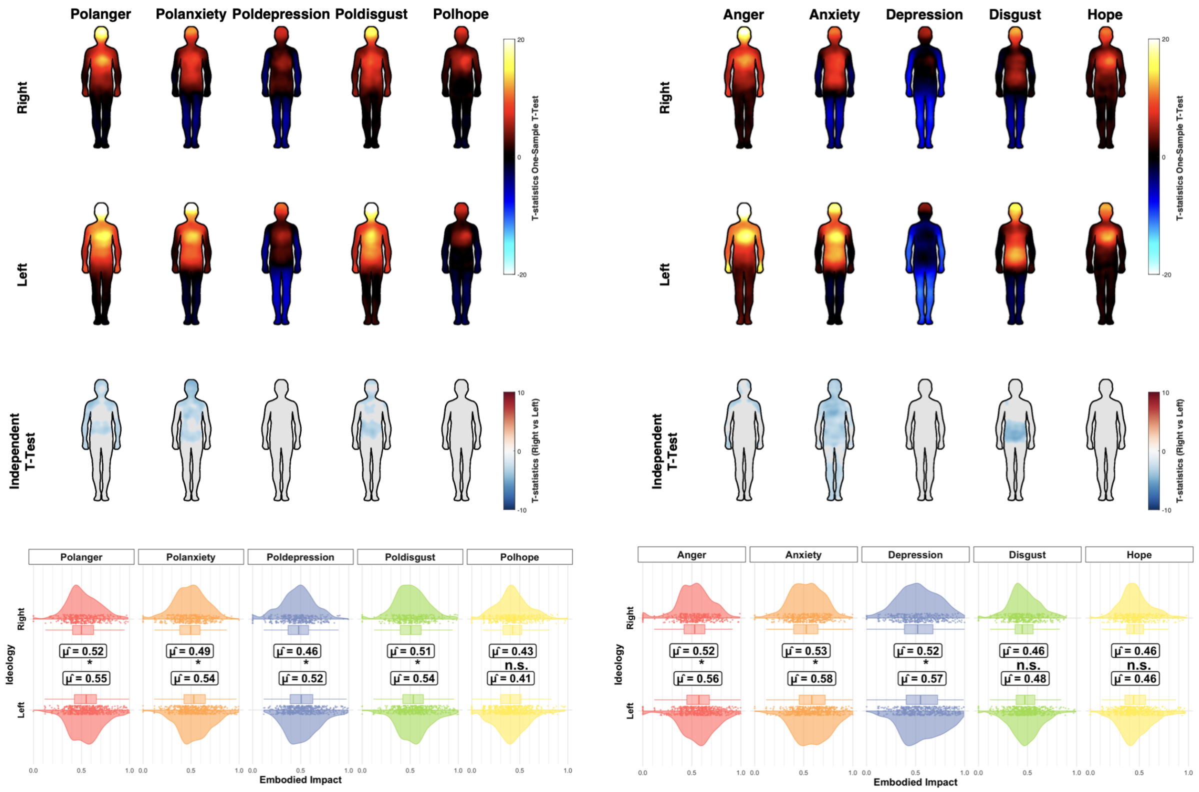


**Fig. S15.** Main Analysis of Partisanship Replaced as Left/Right Ideological Placement (not preregistered).

**Appendix B.3: RQ3.** We report the full GAM specifications for RQ3, including main-effect and interaction models predicting political participation and affective polarization. Consistent with the main text, embodied political impact shows a significant positive association with political participation, whereas embodied non-political impact and self-reported emotional intensity do not. For affective polarization, neither main effects nor preregistered interactions involving embodied impact are significant; polarization is instead primarily explained by political sophistication and partisanship strength. Interaction models indicate that the effects of embodied political impact do not vary meaningfully by sophistication or partisanship. Figure S16 visualizes the partial smooth effects by political emotion.

**Table S5. Main Effects – Political Participation**

**Smooth terms**

| Term | edf | Ref.df | F | p | Sig. |
|---|---|---|---|---|---|
| Political embodied impact (smooth) | 1.855 | 2.385 | 5.311 | .00389 | ** |
| Non-political embodied impact (smooth) | 1.001 | 1.001 | 1.209 | .27179 | |
| Political self-reported intensity (smooth) | 1.001 | 1.001 | 0.013 | .91310 | |

**Parametric terms**

| Term | Estimate | Std..Error | Statistic | p | Sig. |
|---|---|---|---|---|---|
| (Intercept) | 0.589 | 0.173 | 3.408 | < .001 | *** |
| Age | -0.002 | 0.002 | -0.98 | .32715 | |
| Gender (dummy) | 0.244 | 0.066 | 3.676 | < .001 | *** |
| Education (levels) | 0.063 | 0.023 | 2.687 | .00734 | ** |
| Ethnicity (dummy) | -0.031 | 0.07 | -0.434 | .66473 | |
| Political sophistication | 1.854 | 0.175 | 10.573 | < .001 | *** |
| Partisanship strength | -0.076 | 0.011 | -7.014 | < .001 | *** |

*Note.* * p < .05, ** p < .01, *** p < .001. Smooth p-values are approximate. Family: gaussian (link: identity); Method: REML; N = 981; Deviance explained = 19.1%; R2(adj) = 0.183.

**Table S6. Main Effects – Affective Polarization**

**Smooth terms**

| Term | edf | Ref.df | F | p | Sig. |
|---|---|---|---|---|---|
| Political embodied impact (smooth) | 1.65 | 2.086 | 0.74 | .502 | |
| Non-political embodied impact (smooth) | 1.35 | 1.623 | 0.474 | .506 | |
| Political self-reported intensity (smooth) | 1.568 | 1.97 | 1.743 | .207 | |

**Parametric terms**

| Term | Estimate | Std..Error | Statistic | p | Sig. |
|---|---|---|---|---|---|
| (Intercept) | 17.065 | 4.897 | 3.485 | < .001 | *** |
| Age | 0.242 | 0.065 | 3.705 | < .001 | *** |
| Gender (dummy) | 1.847 | 1.882 | 0.981 | .3266 | |
| Education (levels) | -1.468 | 0.661 | -2.22 | .0266 | * |
| Ethnicity (dummy) | 1.101 | 1.994 | 0.552 | .5812 | |
| Political sophistication | 33.055 | 4.964 | 6.658 | < .001 | *** |
| Partisanship strength | -1.066 | 0.305 | -3.494 | < .001 | *** |

*Note.* * p < .05, ** p < .01, *** p < .001. Smooth p-values are approximate. Family: gaussian (link: identity); Method: REML; N = 981; Deviance explained = 9.8%; R2(adj) = 0.088.

## Table S7. Interaction Effect – Political Participation

### Smooth terms

| Term | edf | Ref.df | F | p | Sig. |
|---|---|---|---|---|---|
| Political embodied impact (smooth) | 2.507 | 3.228 | 4.523 | .00384 | ** |
| Political sophistication (smooth) | 1.009 | 1.019 | 110.643 | < .001 | *** |
| Political impact × Political sophistication (interaction) | 3.968 | 5.547 | 0.813 | .56211 | |

### Parametric terms

| Term | Estimate | Std..Error | Statistic | p | Sig. |
|---|---|---|---|---|---|
| (Intercept) | 2.126 | 0.316 | 6.731 | < .001 | *** |
| Non-political impact | -0.37 | 0.335 | -1.102 | .27056 | |
| Political self-reported intensity | 0 | 0.003 | 0.194 | .84650 | |
| Age | -0.002 | 0.002 | -0.957 | .33892 | |
| Gender (dummy) | 0.246 | 0.066 | 3.699 | < .001 | *** |
| Education (levels) | 0.062 | 0.023 | 2.655 | .00807 | ** |
| Ethnicity (dummy) | -0.033 | 0.07 | -0.463 | .64357 | |
| Partisanship strength | -0.076 | 0.011 | -7.078 | < .001 | *** |

*Note.* * $p < .05$, ** $p < .01$, *** $p < .001$. Smooth p-values are approximate. Family: gaussian (link: identity); Method: REML; N = 981; Deviance explained = 20.0%; R2(adj) = 0.188.

## Table S8. Interaction Effect – Affective Polarization

### Smooth terms

| Term | edf | Ref.df | F | p | Sig. |
|---|---|---|---|---|---|
| Political embodied impact (smooth) | 1.001 | 1.002 | 0.639 | .424 | |
| Partisanship strength (smooth) | 6.381 | 7.545 | 72.777 | < .001 | *** |
| Political impact × Partisanship strength (interaction) | 1.008 | 1.016 | 0.182 | .678 | |

### Parametric terms

| Term | Estimate | Std..Error | Statistic | p | Sig. |
|---|---|---|---|---|---|
| (Intercept) | 31.778 | 7.475 | 4.251 | < .001 | *** |
| Non-political impact | 3.966 | 7.681 | 0.516 | .60569 | |
| Political self-reported intensity | 0.044 | 0.059 | 0.751 | .45272 | |
| Age | 0.169 | 0.053 | 3.183 | .00151 | ** |
| Gender (dummy) | -0.83 | 1.518 | -0.547 | .58482 | |
| Education (levels) | -1.679 | 0.533 | -3.151 | .00168 | ** |
| Ethnicity (dummy) | -0.048 | 1.609 | -0.03 | .97609 | |
| Political sophistication | 7.109 | 4.165 | 1.707 | .08817 | |

*Note.* * $p < .05$, ** $p < .01$, *** $p < .001$. Smooth p-values are approximate. Family: gaussian (link: identity); Method: REML; N = 981; Deviance explained = 41.8%; R2(adj) = 0.409.

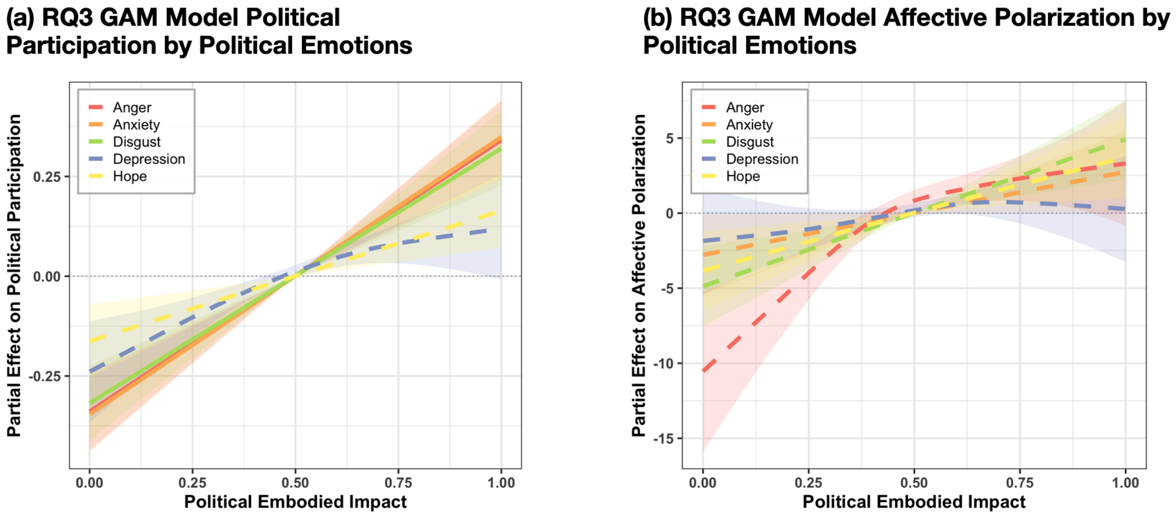


**Fig. S16.** Panel (a) shows the partial effects of political embodied impact on political participation, and Panel (b) shows the effects on affective polarization, each estimated separately for the five political emotions. Solid lines represent significant smooth terms ($p < .05$), while dashed lines indicate non-significant effects. Shaded areas denote 95% confidence intervals. Greater embodied impact was positively associated with higher political participation across political emotions like anger, anxiety and disgust, whereas associations with affective polarization showed weaker and insignificant associations.

## Appendix C: Clarifications and Deviations from the Preregistration

### Changes and Clarifications to the Preregistered Analyses.

- **Control variables.** In the main aggregated and nonlinear analyses, we included a set of sociodemographic control variables (age, gender, education, and Ethnicity) to improve model precision and comparability across specifications. Although these controls were not preregistered, results are substantively identical to the preregistered models. For transparency, the original preregistered models without controls are reported alongside the main results in Appendix B.

- **Equivalence tests (TOST).** As preregistered, equivalence testing was performed using a margin of Cohen's $d = \pm 0.25$. In the preregistration, the TOST was defined in terms of standardized effect sizes and raw group means; however, in the final implementation, the tests were applied to the *model-based estimated marginal means* from the covariate-adjusted ANOVA models. This approach ensures that the equivalence tests reflect the same adjusted estimates used in the main analyses—incorporating control variables and Benjamini–Hochberg adjusted $p$-values—rather than unadjusted sample means. The equivalence bounds were still defined in raw score units ($\pm 0.25 \times$ pooled observed SD), preserving the preregistered effect-size interpretation.

- **Modeling interactions.** For the generalized additive models (GAMs), we used the `ti()` function instead of the preregistered `te()` function to model nonlinear interactions. The `te()` function combines main effects and interactions in a single term, which precludes estimating them separately, whereas `ti()` isolates the pure interaction smooth while retaining independent main-effect smooths. This modification follows current best-practice recommendations for specifying GAM interactions (e.g; 11).

**Additional Analyses.** The most substantial deviation from the preregistered plan was the inclusion of several additional analyses introduced as robustness and diagnostic checks. These analyses, described in the corresponding Results subsections (RQ1–RQ3), were theoretically motivated and intended to test the stability and interpretability of the main findings. None of these additions altered the substantive conclusions of the study.

## References


1. M Tyler, S Iyengar, Testing the Robustness of the ANES Feeling Thermometer Indicators of Affective Polarization. *Am. Polit. Sci. Rev.* **118**, 1570–1576 (2024).
2. G Inc, New High of 45% in U.S. Identify as Political Independents (2026).
3. L Nummenmaa, E Glerean, R Hari, JK Hietanen, Bodily maps of emotions. *Proc. Natl. Acad. Sci.* **111**, 646–651 (2014).
4. L Nummenmaa, R Hari, JK Hietanen, E Glerean, Maps of subjective feelings. *Proc. Natl. Acad. Sci. United States Am.* **115**, 9198–9203 (2018).
5. PR Center, Inflation, health Costs, Partisan Cooperation Among the Nation's Top Problems, Technical report (2023).
6. A Galvez-Pol, M Nadal, JM Kilner, Emotional representations of space vary as a function of peoples' affect and interoceptive sensibility. *Sci. Reports* **11**, 16150 (2021).
7. CS Lloyd, et al., Mapping alexithymia: Level of emotional awareness differentiates emotion-specific somatosensory maps. *Child Abus. & Negl.* **113**, 104919 (2021).
8. LJ Torregrossa, SD Blain, MA Snodgress, S Park, Multidimensional schizotypy and embodied emotions. *Front. Psychol.* **14** (2023).
9. AM Herman, M Wypych, J Michałowski, A Marchewka, Bodily confusion: Lower differentiation of emotional and physiological states in student alcohol users. *Addict. Biol.* **29** (2024).
10. D Lakens, AM Scheel, PM Isager, Equivalence Testing for Psychological Research: A Tutorial. *Adv. Methods Pract. Psychol. Sci.* **1**, 259–269 (2018).
11. U Simonsohn, Interacting With Curves: How to Validly Test and Probe Interactions in the Real (Nonlinear) World. *Adv. Methods Pract. Psychol. Sci.* **7**, 25152459231207787 (2024).
